# A short SUMOylation tag modulates transcription factor activity

**DOI:** 10.1016/j.jbc.2025.110807

**Published:** 2025-10-08

**Authors:** Antoine Y. Bouchard, Anaïs J.I. Vivet, Valérie C. Cabana, Chongyang Li, Pierre Thibault, Marc P. Lussier, Sylvie Mader, Laurent Cappadocia

**Affiliations:** 1 Département de Chimie, Université du Québec à Montréal, Montréal, Québec, Canada; 2 Centre d’Excellence en Recherche sur les Maladies Orphelines – Fondation Courtois (CERMO-FC), Faculté des Sciences, Université du Québec à Montréal, Montréal, Québec, Canada; 3 Regroupement québécois de recherche sur la fonction, l’ingénierie et les applications des protéines (PROTEO), Montréal, Québec, Canada; 4 Institut de Recherche en Immunologie et Cancérologie (IRIC), Université de Montréal, Montréal, Québec, Canada; 5 Département de Chimie, Université de Montréal, Montréal, Québec, Canada; 6 Département de Biochimie et Biologie Moléculaire, Université de Montréal, Montréal, Québec, Canada

**Keywords:** SUMOylation, ZNF451, transcription factor, p53, SUMO E3 ligase, SUMOylation tag, small ubiquitin-like modifier (SUMO)

## Abstract

SUMOylation is a posttranslational modification that regulates multiple aspects of protein biology, including the activity of transcription factors such as p53. Although strategies exist to decrease protein SUMOylation in a targeted manner, options are limited to increase SUMOylation in a protein-specific manner. Here, we developed a strategy to induce SUMOylation of a target protein relying on its genetic fusion to a 32-residue tag termed ZNF and composed of the SUMO E3 module of ZNF451. Through *in vitro* and cell-based assays, we establish that this SUMOylation tag promotes robust poly/multi-SUMOylation of p53, used as a model substrate, with a strong preference for SUMO2/3 as compared to SUMO1. Mass spectrometry experiments performed on transfected HEK293 cells stably expressing a modified form of SUMO3 indicate that lysine 386, the main SUMOylation acceptor site of p53, is the primary target of ZNF-mediated SUMOylation. Increased SUMOylation represses p53 transcriptional activity in luciferase reporter assays, a result compatible with the general repressive effects of SUMOylation on transcription factor activity. Finally, fusion of ZNF to HSF1 and DNMT3A also increase their SUMOylation level, showcasing that ZNF could potentially be used to promote the SUMOylation of a broad range of proteins implicated in DNA metabolism. Overall, this strategy will facilitate the investigation of the impact of increased SUMOylation on specific protein substrates.

Posttranslational modifications (PTMs) play key roles in cell signaling, modulating protein interactions and functions (1). Among PTMs, the ubiquitin-like proteins (UbIs) are a group of small proteins that can be added posttranslationally on a target substrate (2). The best-known Ubl modification, ubiquitination (3, 4), regulates protein stability and degradation *via* the proteasome pathway (5). Targeted ubiquitination can be induced by proteolysis-targeting chimeras (6) that are heterobifunctional small molecules that can specifically bind and degrade a protein of interest through the ubiquitin-proteasome pathway (6, 7). Proteolysis-targeting chimeras are successful examples of how novel molecular tools that exploit PTMs can be utilized for biote therapeutic purposes.

Small-ubiquitin-like modifiers (SUMOs) are other members of the Ubl family that are involved in multiple cellular processes, such as cellular stress responses (8), DNA damage responses (9), protein subcellular localization (10), and transcription regulation (11). SUMOylation consists of the addition of a SUMO protein on target substrates, on lysine residues typically embedded in a SUMOylation consensus motif ψ-K-x-E/D, where ψ is a hydrophobic amino acid, x is any amino acid, and E/D is a glutamic/aspartic acid (12). The ATP-dependent SUMOylation reaction involves the sequential action of three proteins. First, a mature SUMO with an exposed diglycine motif at its C terminus (13) is loaded onto a SUMO-activating (E1) enzyme (SAE1/SAE2 in humans) (14). It is then transferred onto the SUMO conjugating (E2) enzyme (Ubc9 in humans) (15). Finally, SUMO E3 ligases stimulate the transfer of SUMO from the E2 active site to the lysine residue of a substrate (2, 16). SUMO E3 ligases differ by their substrate pools, as some appear specific for certain protein partners (17, 18). In humans, only one SUMO E1, one SUMO E2, and about a dozen SUMO E3s have been discovered (2). The SUMOylation reaction can be reversed by SUMO-specific proteases (SENPs) (19) that recycle SUMO and the substrate in their unconjugated form.

The consequences of adding SUMO on a substrate depend on the type of SUMOylation. Mono-SUMOylation is often associated with the recruitment of protein effectors (20, 21, 22). Poly-SUMOylation (the addition of multiple SUMOs on a single lysine to form a chain) or multi-SUMOylation (the addition of multiple SUMOs on several lysines) can lead to the recruitment of SUMO-targeted ubiquitin ligases (STUbLs), such as RNF4, resulting in target protein ubiquitination and degradation by the proteasome (23). In humans, at least three proteins, SUMO1-3, are ubiquitously expressed in human tissues (24). SUMO2 and SUMO3 share 97% sequence identity, making them indistinguishable in various assays, including immunoblotting. In contrast, SUMO2/3 share only approximately 50% sequence identity with SUMO1. One of the key characteristics distinguishing SUMO2/3 from SUMO1 is their greater capacity to promote poly-SUMOylation (25). Indeed, SUMO2/3 possess a SUMOylation consensus site in their N-terminal region, while this motif is absent in SUMO1. The exact role of SUMOylation also partly depends on the identity of the SUMO protein added to the substrate (25). For instance, SUMO1 has previously been associated with increased solubility of proteins and recruitment of interactors (20, 21), while SUMO2/3 is associated with dynamic processes such as stress responses (26) as well as protein degradation (23). Another key difference between SUMO proteins is their relative cellular abundance: SUMO1 is present in a lesser quantity than SUMO2/3 (24). Also, while the SUMO E1 and E2 enzymes can function with multiple SUMO proteins, certain SUMO E3 ligases, such as ZNF451 (27, 28), display selectivity in SUMO conjugation (29).

The most studied SUMO E3 ligases belong to the SP-RING family, which all contain the characteristic RING-like domain as well as at least one SUMO interactive motif (SIM) (30). Atypical SUMO E3 ligases, including the nuclear pore-associated ligase RanBP2 (31, 32) and the zinc finger protein ZNF451 (27, 28, 33), have also been characterized. ZNF451 possesses the smallest currently known SUMO E3 ligase module. It comprises only two SIMs separated by a PLRP motif for a total size of 3.4 kDa, while the whole protein has a molecular weight of 121 kDa (28). Mechanistically, the first SIM of ZNF451 interacts with E2∼SUMO and maintains the bound SUMO in an active conformation primed for transfer onto a substrate. The second SIM interacts with a second SUMO positioned on the E2 backside. As mentioned before, ZNF451 has a strong bias towards protein conjugation with SUMO2/3 rather than SUMO1, although the exact molecular basis for this SUMO selectivity is still elusive (27, 28).

One of the first proteins reported to be SUMOylated was p53 (34), a tumor suppressor frequently mutated in cancers (35). p53 is a homo-tetrameric nuclear transcription factor that is notably implicated in DNA damage responses (36, 37). It regulates the transcription of many genes involved in cell cycle arrest, senescence, and apoptosis, such as p21 and Bax (38, 39, 40). p53 can be modified by SUMO1 or SUMO2/3 under different conditions (41, 42). Although SUMO1 is mostly added to p53 under normal cell conditions, SUMO2/3 is typically added during cellular stress, leading to p53-induced apoptosis or senescence (41, 42, 43, 44). Although the specific effects of these modifications remain somewhat elusive, most studies suggest that p53 SUMOylation influences its transcriptional activity (42, 45), with possible differential effects between SUMO1 and SUMO2.

Although strategies exist to decrease SUMOylation, options are limited to increase SUMOylation. Most strategies involve mutating SUMOylation sites, underexpressing or overexpressing components of the SUMO machinery, or fusing SUMO at the N or C terminal extremity of the protein of interest to mimic protein SUMOylation (41, 46, 47, 48). All these approaches present significant limitations, either affecting the interplay with other PTMs, affecting other protein substrates, or not accurately mimicking the modification of a lysine residue by SUMO. In addition, it is difficult to control the type of SUMOylation added when modulating the expression of SUMOylation enzymes. An interesting approach developed several years ago involves fusing the SUMO E2 to the protein of interest to increase its SUMOylation (46). This promotes the SUMOylation of the fused substrate by increasing local concentrations of E2 activity, thus providing a valuable approach to study the consequences associated with increased SUMOylation. However, increasing E2 concentrations in cells, perhaps at noncognate locations, could impact global SUMOylation levels due to its high activity, as this sole E2 is responsible for all the SUMOylation happening in the cell. In addition, this approach is not specific to a single SUMO protein type.

Here, we describe a new strategy to specifically increase SUMOylation of a substrate relying on the fusion of the N-terminal fragment of ZNF451 responsible for its SUMO E3 ligase activity to a protein target. We provide proof of concept by demonstrating that this approach efficiently increases the SUMOylation of a fused p53 protein compared to the unfused p53 both *in vitro* and in transfected cells. As expected, the SUMOylation reaction showed a selective preference for SUMO2/3 over SUMO1. Mass spectrometry experiments performed on transfected HEK293 cells stably expressing a modified form of SUMO3 facilitating purification and identification of SUMOylated peptides indicated that the ZNF451 fragment promoted SUMOylation of p53 on lysine 386, the canonical p53 SUMOylation site (49). Mutating one of the SIMs of the ZNF451 fragment limited the extent of induced SUMOylation, enabling a fine-tuning of SUMOylation levels. Although we saw no signs of preferential degradation of SUMOylated species, SUMOylation reduced the transcription activity of p53 in a reporter assay in a manner that was dependent on SUMO E1 activity. Finally, we report that fusing the ZNF451 fragment to heat shock factor 1 (HSF1) and DNA methyltransferase 3A (DNMT3A), two proteins involved in gene transcription, also increases their SUMOylation, thus indicating that this strategy could potentially be applied to a wide range of proteins. Overall, our results demonstrate that our ZNF451-derived tag is efficient at inducing SUMO2/3 modification of a target protein, and that this strategy can be used to modulate transcription factor activity.

## Results

### Fusion of the ZNF451 SUMO E3 module with p53 increases its SUMOylation *in vitro*

**Figure 1:**
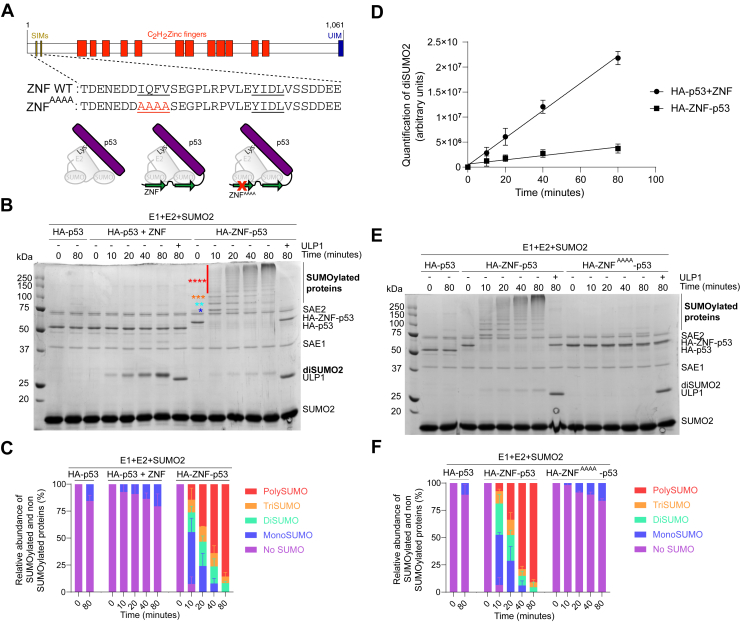
**A ZNF-p53 fusion increases p53 SUMOylation with SUMO2.***A*, *top*: full ZNF451 protein, with the main domains identified. ZNF451 contains 2 SUMO-interacting motifs (SIMs), 12 C_2_H_2_ Zinc Fingers, and a ubiquitin-interacting motif (UIM). *Middle*: the complete sequence of WT ZNF and ZNF^AAAA^ tags used in this study. *Bottom*: pictograms illustrating p53 (in *purple*) either not fused (*left*) or fused (*right*) with ZNF, the SUMO E3 module of WT ZNF451 (*green arrows*), and the SUMO-charged E2 (in *gray*). *B*, *E*, *in vitro* assays comparing the SUMOylation of HA-p53, HA-p53+ZNF, and HA-ZNF-p53 (*B*) or of HA-p53, HA-ZNF-p53, and HA-ZNF ^AAAA^-p53 (*E*) in the presence of E1 (SAE1 and SAE2), E2 (not visible on gel), and SUMO2. Reactions were started by adding 2 mM ATP and stopped at the indicated time points by the addition of Laemmli buffer. For ULP1-treated samples, reactions were allowed to proceed for 80 min, then 1 μM ULP1 was added for 10 min. Reactions were loaded on a 12% SDS-PAGE and colored with *Coomassie blue*. *Stars* represent SUMOylation states (∗ mono-SUMOylation, ∗∗ di-SUMOylation, ∗∗∗ tri-SUMOylation, and ∗∗∗∗ poly-SUMOylation). Gels are representative of three independent experiments (N = 3). *C*–*F*, relative abundance of SUMOylated and non-SUMOylated p53 determined by quantification of band intensities for the conditions presented in *B* or *E*. Detailed quantifications are shown on Figs. S1 and S2, respectively. *D*, quantification of the intensity of the band corresponding to diSUMO2 forms of HA-ZNF-p53 and HA-p53+ZNF in *B*.

The N-terminal region of ZNF451 displays robust *in vitro* SUMO E3 activity (27, 28). To induce the SUMOylation of p53, we generated a fusion protein between p53 and a fragment of ZNF451 containing residues 24 to 55, hereafter called ZNF (Fig. 1*A*). The small size of this fragment (32 residues) likely minimizes steric impact on the attached proteins. In addition, we added an N-terminal hemagglutinin (HA) tag to facilitate immune detection. Importantly, neither the HA-tag, the linkers, nor ZNF contain lysine residues and, therefore, cannot be targeted by SUMOylation. To assess the capacity of fused ZNF to increase the SUMOylation of p53, we first used *in vitro* assays and compared the SUMOylation levels of HA-ZNF-p53 (also referred as ZNF *in cis*) to those of HA-p53 either alone or in the presence of the ZNF fragment added *in trans* (HA-ZNF + p53, Fig. 1*B*). The HA-ZNF-p53 fusion underwent rapid and robust SUMOylation, characterized by a decrease in the abundance of the unmodified HA-ZNF-p53 and concomitant appearance and accumulation of higher molecular weight species corresponding to SUMO-modified p53. The addition of the SUMO protease ULP1 after 80 min fully reversed the modifications, thus confirming that the higher molecular weight species detected genuinely correspond to SUMOylated species (Fig. 1*B*). Indeed, after ULP1 treatment, the amounts of unmodified HA-ZNF-p53 increased while higher molecular weight species disappeared, confirming that the fusion protein undergoes SUMOylation. Taken together, these results suggest that the HA-ZNF-p53 fusion undergoes rapid SUMOylation.

To characterize the rate of SUMOylation, we quantified the distribution of mono-SUMOylated (∗), di-SUMOylated (∗∗), tri-SUMOylated (∗∗∗), and poly/multi-SUMOylated (four or more SUMOs, ∗∗∗∗) states at each time point (Figs. 1*C* and S1). The band corresponding to the unmodified HA-ZNF-p53 decreased to less than 10% of the total species after 10 min and was undetectable after 20 min. The amount of mono-SUMOylated fusion peaked at 10 min with a significant difference observed between HA-ZNF-p53 and HA-p53 + ZNF (Fig. S1), before rapidly decreasing. Di- and tri-SUMOylated species abundance stayed relatively constant throughout the reaction, while levels of poly/multi-SUMOylated proteins increased over time, becoming the predominant forms at 80 min. In sharp contrast to the HA-ZNF-p53 fusion, only unmodified and mono-SUMOylated p53 was observed after 80 min for the unfused protein in the absence or presence of ZNF added in *trans*. Taken together, these results indicate that fusing ZNF to p53 sharply increases its SUMOylation kinetics.

Although we did not observe a substantial increase in the SUMOylation of p53 when ZNF is added in *trans*, we did notice the accumulation of diSUMO2 (identified in bold in Fig. 1*B*). Since diSUMO2 is barely detectable in the absence of ZNF, while apparent in conditions where ZNF is present, we suspected that the formation of diSUMO2 is due to the SUMO E3 ligase activity of ZNF that utilizes SUMO2 as a substrate, as previously reported for ZNF451 (27). Furthermore, when comparing the band intensity of diSUMO2 when ZNF is used in *trans* or in *cis*, we noticed a 5-time greater accumulation of diSUMO2 for reactions using HA-p53+ZNF compared to reactions using HA-ZNF-p53 (Fig. 1*D*), suggesting that the tethered substrate p53 reduces the activity of ZNF toward other substrates. This can be explained by the fact that, although free SUMO concentration is considerably higher than unfused p53 in the *in vitro* assays, forced proximity of ZNF with p53 selectively favors SUMOylation *via* an intramolecular reaction.

### Mutation of the first SIM motif in ZNF reduces p53 SUMOylation

To further explore the mechanism of p53 SUMOylation by the fused ZNF, we mutated the first SIM of ZNF451 to decrease its interaction with the SUMO-charged E2 (Fig. 1*A*). Compared to the fusion with wild-type ZNF, we observed a substantial decrease in p53 SUMOylation when fused to the ZNF^AAAA^ mutant in our *in vitro* assays (Fig. 1*E*). Indeed, while HA-ZNF-p53 rapidly reached a poly-SUMOylated state, no poly-SUMOylation and barely any di- or tri-SUMOylation could be detected for the HA-ZNF^AAAA^-p53 mutant, even after 80 min (Figs. 1*F* and S2). Mono-SUMOylation levels for this protein were similar to those of the HA-p53 control, suggesting that altering the first SIM of ZNF effectively suppresses its SUMO E3 ligase activity *in vitro*. The fact that little diSUMO2 formation could be observed in the presence of HA-ZNF^AAAA^-p53 is further consistent with a strong decrease in the SUMO E3 activity of the mutated ZNF (Fig. 1*E*). Overall, these observations align with previous reports suggesting that both SIMs are essential for robust SUMO E3 activity of ZNF451 (27, 28). These results further support the conclusion that the induced SUMOylation of HA-ZNF-p53 is due to the SUMO E3 activity of the ZNF fragment.

### ZNF maintains a selectivity for SUMO2 in the context of the HA-ZNF-p53 fusion

**Figure 2:**
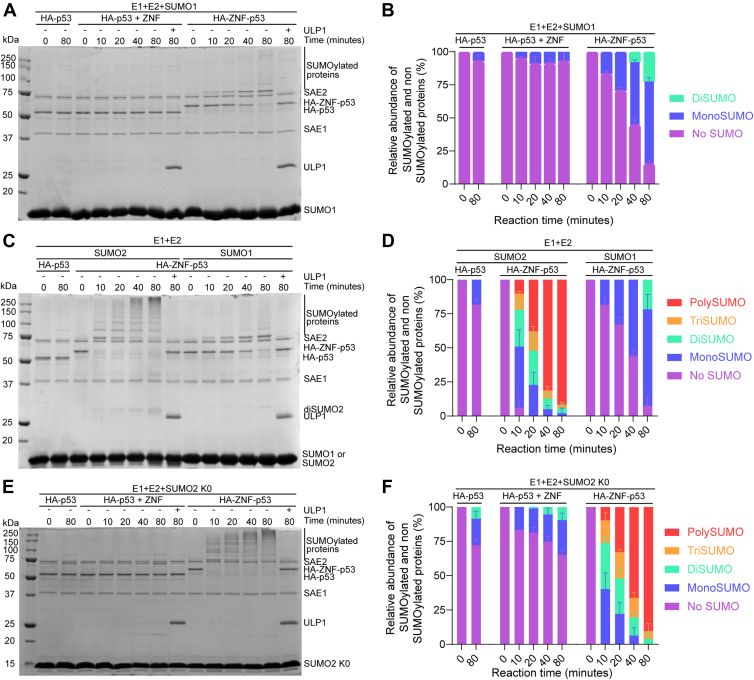
**ZNF SUMO E3 ligase displays less activity with SUMO1 and promotes poly/multi SUMOylation of HA-ZNF-p53.***A*, *C*, and *E*, *in vitro* assays comparing the SUMOylation of *A*, HA-p53 in the presence of ZNF (HA-p53 + ZNF) and the HA-ZNF-p53 fusion using SUMO1, *C*, HA-ZNF-p53 using SUMO1 or SUMO2, and *E*, HA-ZNF-p53 or HA-p53+ZNF using SUMO2 K0. In all cases, HA-p53 was used as a control for basal SUMOylation. Reactions were performed as in Figure 1. Representative *Coomassie blue-stained* 12% SDS-polyacrylamide gels from three independent experiments (N = 3) are shown. *B*, *D*, and *F*, relative abundance of SUMOylated and non-SUMOylated p53 determined by quantification of the band intensities for the conditions presented in *A*, *C*, and *E*, respectively. Detailed quantifications are shown in Fig. S3, S4 and S6.

Previous reports indicate that ZNF451 has a strong preference for SUMO2 rather than SUMO1 (27, 28). To assess whether the ZNF module displays the same SUMO type preference in the context of the HA-ZNF-p53 fusion, we performed *in vitro* assays using SUMO1 instead of SUMO2 (Fig. 2*A*). Although mono-SUMOylation and di-SUMOylation of HA-ZNF-p53 increased rapidly with time with SUMO2 (Fig. 1*C*), modification of the fusion protein by SUMO1 proceeded at a slower pace and only reached mono-SUMOylation, the main SUMOylation product, at 80 min (Figs. 2*B* and S3). SUMOylation of HA-ZNF-p53 was significantly increased compared to HA-p53 + ZNF, beginning at 10 min. Also, contrasting with reactions performed using SUMO2, little diSUMO was detected with SUMO1. This was expected since SUMO1 forms chains less readily due to the absence of the consensus SUMOylation site on SUMO2 (50). Accordingly, the di-SUMOylation of HA-ZNF-p53 may correspond to multi-SUMOylation of p53 rather than poly-SUMOylation. A side-by-side comparison of HA-ZNF-p53 SUMOylation by SUMO1 *versus* SUMO2 (Figs. 2, *C*, *D* and S4) confirmed that the modification rate is significantly faster with SUMO2 than with SUMO1, as evidenced by the rapid disappearance of the band corresponding to the un-SUMOylated substrate only for SUMO2. The highest levels of mono-SUMOylation were detected after 10 min for SUMO2 but after 80 min for SUMO1 (Figs. 2*D* and S4). As before, we verified that the SUMOylation of HA-ZNF-p53 depends on the presence of two intact SIMs by mutating SIM1 of ZNF in fusion (Fig. S5*A*). Similar to experiments performed with SUMO2 (Fig. 1*F*), HA-ZNF^AAAA^-p53 showed a strong decrease in SUMOylation levels compared to the wild-type fusion (Fig. S5*B*). Taken together, our results suggest that the ZNF module acts as a SUMO E3 ligase with a preference for SUMO2 in the context of the HA-ZNF-p53 fusion.

### ZNF can promote SUMOylation at multiple p53 residues *in vitro*

Our previous experiments did not discriminate between multi-SUMOylation (multiple lysine residues are modified with SUMO2), poly-SUMOylation (progressive building of a SUMO chain on a single lysine of the substrate) or both poly- and multi-SUMOylation of p53. To differentiate between these possibilities, we used a mutant of SUMO2, hereafter called SUMO2 K0, in which all lysine residues have been replaced with arginine residues, thereby inhibiting the formation of SUMO chains and preventing the poly-SUMOylation of p53. In assays performed in the presence of SUMO2 K0 and comparing the kinetics of the reactions involving HA-ZNF-p53, HA-p53+ZNF, and HA-p53 alone, we observed a rapid apparition of bands with several SUMOs attached and the concomitant disappearance of unmodified HA-ZNF-p53 within the first 10 min of the reaction (Figs. 2, *E*, *F* and S6). The multiplicity of bands observed with SUMO2 K0 and similar reaction speed as wild-type SUMO2 (compare Figs. 1*B* and 2*E*) suggests that several residues can be targeted on the substrate protein. However, we noticed different migration patterns on the gel. Indeed, whereas clear and regularly spaced bands were observed when using wild-type SUMO2 (Fig. 1*B*), the ladder pattern was more irregularly spaced and clustered when using SUMO2 K0 (Fig. 2*E*). As migration on SDS-polyacrylamide gels can be altered by the shape of proteins, our data hint toward poly-SUMOylated chains being formed with SUMO2 WT but not with SUMO2 K0. Nevertheless, these analyses do not rule out the possibility of multi-SUMOylation of p53 with SUMO2 WT.

### Mutation of the main SUMOylation acceptor site of p53 reduces the kinetics of mono-SUMOylation

**Figure 3:**
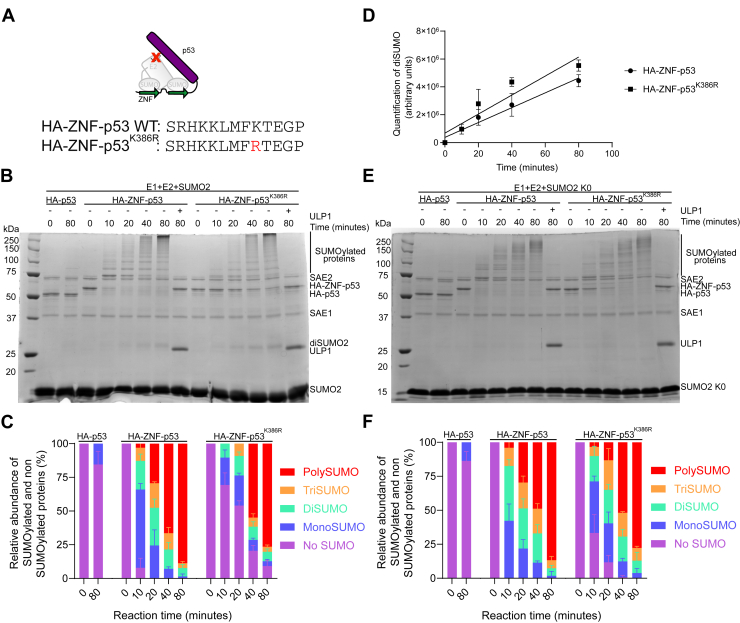
**Mutation of the main SUMOylation acceptor site of HA-ZNF-p53 delays the monoSUMOylation of p53 but does not affect its poly/multi-SUMOylation.***A*, pictogram illustrating the mutation and sequence of p53 and p53^K386R^. *B*–*E*, *in vitro* assays comparing the SUMOylation of HA-p53, HA-ZNF-p53, and HA-ZNF-p53^K386R^ in the presence of E1, E2, and SUMO2 (*B*) or SUMO2 K0 (*E*). Reactions were performed as in Figure 1. Gels are representative of three independent experiments (N = 3). *C*–*F*, relative abundance of SUMOylated and non-SUMOylated p53 determined by quantification of the band intensities for the conditions presented in *B*–*E*. Detailed quantifications are shown on Fig. S7, and S8. *D*, quantification of the intensity of the band corresponding to diSUMO2 in HA-ZNF-p53 and HA-ZNF-p53^K386R^ conditions.

Although K386 was reported as the principal SUMOylation site of p53 (49), the protein still contains 19 other lysine residues, including multiple ubiquitination sites (51). To confirm that lysine 386 of p53 remains the preferred SUMOylation site for ZNF-based SUMOylation, we mutated this residue into an arginine (K386R) and compared its SUMOylation levels to the one of wild-type HA-ZNF-p53 (Fig. 3*A*). In both cases, clear and regularly spaced bands of high molecular weight were visible through time for both fusion proteins (Fig. 3*B*). Although the wild-type fusion was almost entirely SUMOylated after 10 min, a significantly higher amount of the K386R mutant remained un-SUMOylated from 10 to 40 min (Figs. 3*C* and S7). This suggests that K386 is the preferred site of SUMOylation, but that secondary lysine(s) can also be SUMOylated *in vitro*. At the 10 min time-point, the mono-SUMOylation peak represented 60% of all species for the wild-type fusion (60%) compared to only 20% for the K386R mutant, suggesting a faster SUMOylation of K386 compared to other sites. A significant difference was also observed when comparing the percentages of di- and tri-SUMOylated proteins in the earlier stages of the reactions. For poly/multi-SUMOylations, while a significant difference was observed at the 20 min time point in favor of HA-ZNF-p53 compared to K386R mutant, SUMOylation levels were comparable for the rest of the reactions. Interestingly, the similar profiles of di-, tri-, and poly/multi-SUMOylation suggest that the rate of modification is not affected by the K386R mutation. Levels of diSUMO2 remained similar for both fusion proteins, indicating otherwise comparable activity of the ZNF tag in both fusions (Fig. 3*D*). Finally, to assess whether the K386R mutant can be multi-SUMOylated, we performed experiments using SUMO2 K0 (Fig. 3*E*). While we observed that the rate of SUMOylation of the K386R mutant was significantly slower than that of the wild-type protein at the 10 and 20 min time points, no significant differences were observed for the different SUMOylation stages at other time points with SUMO2 K0, except at 10 min for di-SUMOylation (Figs. 3*F* and S8). Altogether, these results confirm that K386 is the preferred site of SUMOylation of p53 while also highlighting the capacity of the fused ZNF to bypass the need for a strong acceptor site and effectively achieve robust substrate poly/multi-SUMOylation.

### Fusion of ZNF to p53 increases its modification with SUMO2/3 in transfected cells

**Figure 4:**
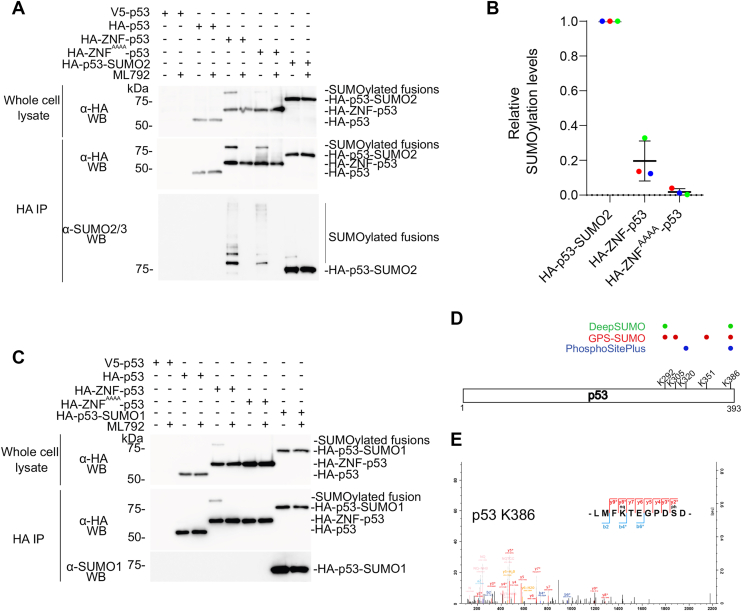
**Fusion of ZNF to p53 increases its SUMOylation with SUMO2 but little with SUMO1 in HEK293 cells.***A*, immunoblot analysis of whole cell lysates or immunoprecipitated HA-p53, HA-ZNF-p53, HA-ZNF^AAAA^-p53, or HA-p53-SUMO2 following expression in HEK293 cells for 48 h. HEK293 cells were treated 4 h with ML792 or control (0.01% DMSO) then lysed. Immunoprecipitation assays were performed overnight at 4 °C using anti-HA antibodies, followed by washes and elution in Laemmli buffer. Proteins were separated on a 7.5% SDS-PAGE. Immunoblots were performed using anti-HA antibodies on whole cell lysates and anti-HA or anti-SUMO2/3 antibodies on immunoprecipitated proteins. *B*, relative SUMOylation levels for HA-ZNF-p53 and HA-ZNF^AAAA^-p53 were obtained from anti-HA and anti-SUMO2/3 immunoblots presented in *A* after normalization with HA-p53-SUMO2 for which association between p53 to SUMO2 is equimolar. *C*, immunoblot analysis of immunoprecipitated HA-p53, HA-ZNF-p53, HA-ZNF^AAAA^-p53, or HA-p53-SUMO1 following expression in HEK293 cells with detection using anti-HA or anti-SUMO1 antibodies. Representative immunoblots from three independent experiments (N = 3) are shown. *D*, identification of potentials SUMOylation sites in p53 using PhosphoSitePlus (*blue*), GPS-SUMO (*red*), and DeepSUMO (*green*). Confirmed (PhosphoSitePlus) or high-confidence (GPS-SUMO and DeepSUMO) predictions are represented with colored dots. *E*, mass spectrum of HA-ZNF-p53 transfected in HEK293 cells stably expressing His_6_-SUMO3^Q87R/Q88N^ and showing SUMOylation on lysine 386 and phosphorylation on Ser392. DMSO, dimethyl sulfoxide; HA, hemagglutinin.

Following the characterization of the HA-ZNF-p53 fusion *in vitro*, we assess the capacity of the ZNF tag to induce p53 SUMOylation in a fusion protein in a model human cell line. We thus transfected V5-p53, HA-p53, HA-ZNF-p53, HA-ZNF^AAAA^-p53, and HA-p53-SUMO2 as a positive control for the detection of SUMO-fused p53, in HEK293 cells for 48 h and analyzed their expression and SUMOylation (Fig. 4*A*). In anti-HA immunoblots of whole cell lysates, HA-p53, HA-ZNF-p53, and HA-ZNF^AAAA^-p53 accumulated to similar levels. Although no SUMOylation event could be readily detected for HA-p53, a band corresponding to mono-SUMOylation was detected with HA-ZNF-p53 and was noticeably more intense compared with HA-ZNF^AAAA^-p53. Following HA immunoprecipitation, we confirmed that SUMOylation of HA-ZNF-p53 was strongly increased compared to HA-p53 using an anti-SUMO2/3 antibody. In line with our *in vitro* assays, SUMOylation bands associated with HA-ZNF-p53 were also more intense than those for HA-ZNF^AAAA^-p53. However, in contrast with our *in vitro* assays, HA-ZNF^AAAA^-p53 SUMOylation was much more pronounced than the HA-p53 control. To quantify the percentage of HA-ZNF-p53 undergoing SUMOylation, we normalized the SUMOylation signals of both fusion proteins with HA-p53-SUMO2 where association between p53 to SUMO2 is equimolar (Fig. 4*B*). This analysis suggests that around 20% of HA-ZNF-p53 is SUMOylated, while around 3% of the AAAA mutant is SUMOylated. We conclude that ZNF promotes the SUMOylation of fused p53 by SUMO2/3 in HEK293 cells in a manner that is partially dependent on the first SIM.

To confirm that the SUMOylation increase is dependent on the SUMOylation machinery, we treated transfected cells with ML792, a SUMO E1 inhibitor (52). In comparison to vehicle-treated samples, SUMOylation was completely abolished in samples treated with ML792 in anti-HA or anti-SUMO2/3 immunoblots after immunoprecipitation with anti-HA antibodies (Fig. 4*A*), confirming that the visible high-molecular-weight bands correspond to SUMOylated proteins. In contrast, detection of SUMO2-fused HA-p53-SUMO2 was, as expected, not affected by ML792 in the SUMO2/3-immunoblots (Fig. 4*A*). The intensity of the band associated to HA-p53-SUMO2 was stronger in the anti-SUMO2/3 immunoblot compared to that of HA-ZNF-p53 and HA-ZNF^AAAA^-p53, a result compatible with a fraction of HA-ZNF-p53 and HA-ZNF^AAAA^-p53 being SUMOylated. Overall, these results indicate that the increased SUMOylation caused by fusing ZNF to p53 is dependent on the activity of the endogenous SUMOylation cascade.

Our *in vitro* assays suggest a strong preference of HA-ZNF-p53 for SUMO2 compared to SUMO1. To test whether a similar outcome for SUMO1 would be observed in a cellular context, we repeated our immunoprecipitation assays using an anti-SUMO1 antibody to detect SUMO1-modified proteins, and SUMO1-fused p53 as a positive control (Fig. 4*C*). Although the anti-SUMO1 antibody readily detected the HA-p53-SUMO1 control upon immunoprecipitation with the anti-HA antibody, no SUMO1-modified proteins were detected in cells transfected with HA-ZNF-p53, HA-p53 or HA-ZNF^AAAA^-p53. Higher molecular bands likely corresponding to SUMO2/3 modification of HA-ZNF-p53 were still detectable when using the anti-HA antibody. Overall, these experiments suggest that fusing ZNF to p53 results in preferential modification of p53 by SUMO2/3 compared to SUMO1 in a cellular context, similar to what was observed *in vitro*.

### ZNF induces p53 SUMOylation mainly at its canonical K386 site in transfected cells

Our *in vitro* experiments suggest that ZNF can promote poly-SUMOylation and/or multi-SUMOylation when fused to a substrate. To further identify SUMOylation sites on p53 and SUMO2, we analyzed SUMOylation reactions performed *in vitro* using HA-p53, HA-ZNF-p53, or HA-ZNF-p53^K386R^ and either His_6_-SUMO2^Q8^^8^^R^ or His_6_-SUMO2^Q8^^8^^R^ K0 (Fig. S9*A*). The Q88R mutant of SUMO2 was used to facilitate the identification of SUMOylated peptides through mass spectrometry. Reactions were stopped after 10 min and mass spectrometry identified QQTGG adducts on SUMO2 residues 11, 33, and 45, indicating chain formation involving those residues (Fig. S9*B*). Precisely, chains involving lysine 45 of SUMO2 were only detected in ZNF-containing samples whereas ZNF increased the abundance of chains involving K11 and K33 by a factor of 8 and 2, respectively. Although modification of HA-p53 with His_6_-SUMO2^Q8^^8^^R^ was only detected on K386 (Fig. S9*C*), additional SUMOylation sites—K120, K320, K321, and K357—were identified when HA-ZNF-p53 was used. Using the His_6_-SUMO2^Q8^^8^^R^ K0 mutant, a total of three SUMOylation sites (K320, K321, and K386) were detected on HA-p53, while thirteen sites (K120, K132, K139, K164, K291, K292, K319, K320, K321, K357, K373, K382, and K386) were identified on HA-ZNF-p53. These SUMOylation sites partially overlap with those reported on PhophoSitePlus or predicted using DeepSUMO and GPS-SUMO (Fig. 4*D*). In addition, our analyses confirm the formation of SUMO2 chains using lysine residues 11, 33, and 45. The results also suggest that inhibition of SUMO chain formation, *via* the His_6_-SUMO2^Q8^^8^^R^ K0 mutant, expands the repertoire of lysine residues undergoing SUMOylation while lysine 386 consistently appears as a major SUMOylation site for p53, both in presence and absence of ZNF.

To identify ZNF-induced p53 SUMOylation sites in a cellular context, we transfected HA-p53 and HA-ZNF-p53 in HEK293 cells stably expressing His_6_-SUMO3^Q87R/Q88N^. Only one SUMOylation site, lysine 386, corresponding to the major SUMOylation for wild-type p53 (45, 49), was detected exclusively for HA-ZNF-p53, while no SUMOylation was detected on HA-p53 (Fig. 4*E*). Although we did not obtain evidence of SUMO-chain formation, we did notice concomitant SUMOylation at lysine 386 and phosphorylation at serine 392 for HA-ZNF-p53, thus suggesting a possible interplay between these PTMs.

### SIM1 of ZNF contributes to full SUMOylation activity in transfected cells

**Figure 5:**
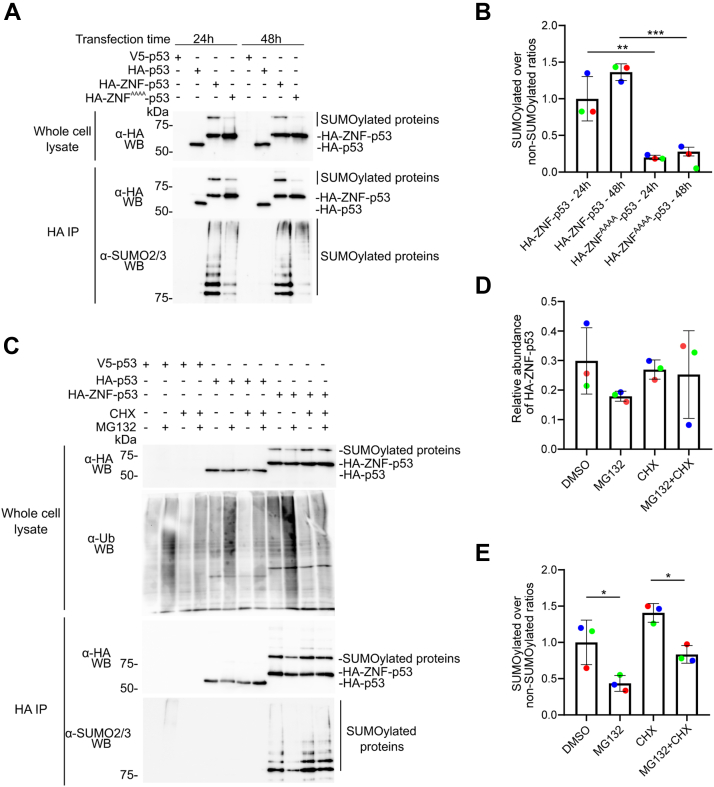
**ZNF-mediated SUMOylation of p53 does not promote strong degradation by the proteasome.***A*–*C*, immunoblot analysis of immunoprecipitated HA-p53, HA-ZNF-p53, or HA-ZNF^AAAA^-p53 from whole cell extracts of HEK293 cells transfected for 24 or 48 h (*A*), or with HA-p53 or HA-ZNF-p53 for 48 h followed by treatment with cycloheximide (CHX) or control (DMSO 0.01%) for 30 min, then with MG132 or control (DMSO 0.01%) for 4 h (*C*). Proteins were separated on a 7.5% SDS-PAGE and immunoblots were performed using anti-HA or anti-SUMO2/3 antibodies. Representative immunoblots from three independent experiments (N = 3) are shown. *B*–*E*, quantification of SUMOylation levels was performed by measuring the band intensities for each condition of immunoprecipitated proteins and is presented relative to the control treatment. *D*, relative abundance of HA-ZNF-p53 was obtained by dividing the band intensities for each condition in the whole cell lysate immunoblot by the total band intensity. Normality was assessed using the Shapiro-Wilk test. A one-way ANOVA statistical analysis, followed by Tukey’s multiple comparison test, was performed to compare transfection conditions within the same treatment (∗ = *p* < 0.05, ∗∗ = *p* < 0.01, ∗∗∗ = *p* < 0.001, ∗∗∗∗ = *p* < 0.0001). For SUMOylation quantification, SUMOylated over non-SUMOylated protein ratios were obtained by dividing the relative band intensity measured in anti-SUMO2/3 immunoblot by the relative band intensity measured in the HA immunoblots. DMSO, dimethyl sulfoxide; HA, hemagglutinin.

To assess the accumulation of SUMOylation at an earlier time, we analyzed the expression and SUMOylation of our constructions in HEK293 cells 24 h as well as 48 h post transfection (Fig. 5*A*). In anti-HA immunoblots of whole cell lysates, the bands corresponding to un-SUMOylated or mono-SUMOylation proteins accumulated to similar levels after 24 and 48 h of transfection, both for HA-ZNF-p53 and HA-ZNF^AAAA^-p53. Quantifying the total SUMOylation in the anti-SUMO2/3 immunoblots and normalization with the amount of immunoprecipitated protein visible in the anti-HA immunoblots suggested that the global proportion of SUMOylated proteins was slightly higher at 48 h, although the increase is statistically nonsignificant (Fig. 5*B*). Interestingly, the results show a statistically significant difference in SUMOylation levels between HA-ZNF-p53 and HA-ZNF^AAAA^-p53, both for 24- and 48-h (Fig. 5*B*). Consistent with our prior observations (Fig. 4*B*), we observed a ∼10 fold decrease in the SUMOylation levels of HA-ZNF^AAAA^-p53 compared to those of HA-ZNF-p53, thus confirming the importance of intact ZNF for full SUMOylation activity *in cellula*.

### ZNF-promoted SUMOylation of p53 does not lead to significant increases in proteasomal degradation

Protein poly-SUMOylation using SUMO2/3 can lead to ubiquitination of the SUMOylated proteins by STUbLs such as RNF4 or RNF111, which ultimately triggers protein degradation by the proteasome (53). To detect possible changes in the levels of poly-SUMOylated p53, we inhibited proteasome activity using MG132 treatment for 4 h to allow the accumulation of proteins normally degraded by the ubiquitin-proteasome system, such as p53, while avoiding cell stress associated with long-term treatment. MG132 treatment efficacy was confirmed by the accumulation in global protein ubiquitination detected using the anti-ubiquitin P4D1 antibody. Quantification of HA-tagged fusion proteins in whole cell lysates revealed that the treatments did not significantly impact protein expression (Fig. 5, *C* and *D*). Following immunoprecipitation of the HA-tagged proteins, a statistically significant reduction in the amounts of SUMOylated HA-ZNF-p53 protein was observed in MG132-treated cells compared to control cells (Fig. 5, *C* and *E*). To better understand how proteasome inhibition could lead to a smaller proportion of SUMOylated HA-ZNF-p53, we quantify the levels of free SUMO2/3 by performing immunoblots using an anti-SUMO2/3 antibody on the whole cell lysate of untreated cells, or cells treated with either dimethyl sulfoxide (DMSO) or MG132 (Fig. S10). These immunoblots reveal that a significantly smaller quantity of free SUMO2/3 is available in cells treated with MG132, raising the possibility that the SUMOylation of HA-ZNF-p53—which requires SUMO2 binding on the E2 backside—may be more dependent on the availability of free SUMO2/3. We also noticed a significant decrease in SUMOylation for the combined cycloheximide (CHX) + MG132 treatment compared to CHX alone. In contrast, no significant difference was observed between CHX treatment and control. The HA-ZNF-p53 SUMOylation was similar for both controls and cells pretreated with the protein cycloheximide (54) for 30 min before MG132 treatment (Fig. 5*D*), indicating that SUMOylation of p53 is stable through the lifetime of the protein and does not significantly undergo increased degradation by the proteasome.

### SUMOylation of p53 represses its transcriptional activity in reporter assays

**Figure 6:**
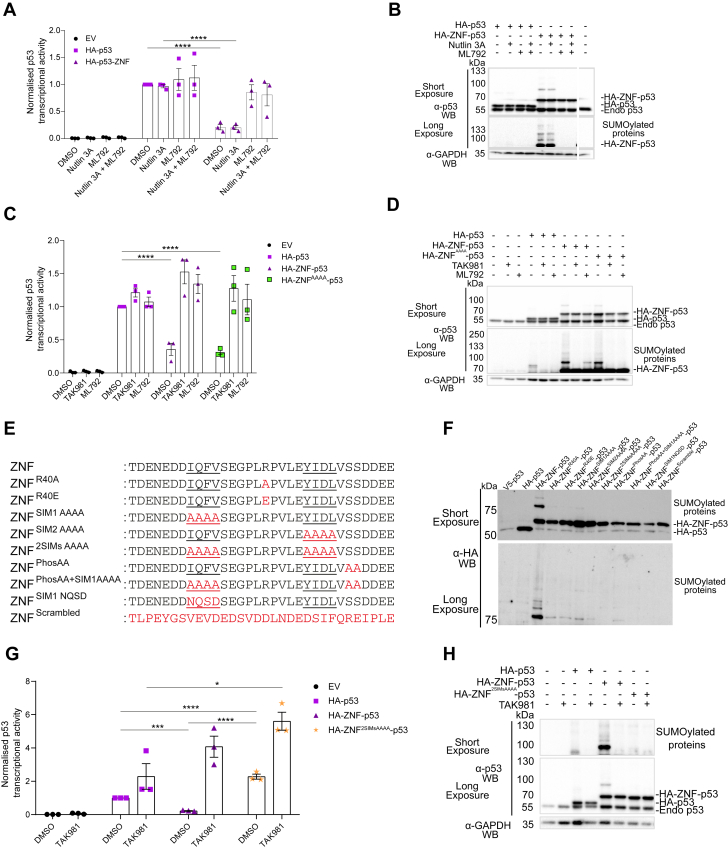
**Targeted SUMOylation of p53 diminishes its transcriptional activity.***A*, *C*, and *G*, the transcriptional activities of ectopically expressed p53 fusion proteins were assessed using the p53 luciferase reporter vector pG13-luc. Luciferase activity was measured after HEK293 cell transfection for 48 h with indicated expression vectors for p53, ZNF-p53, ZNF^AAAA^-p53. ZNF^2SIMSAAAA^-p53 or the parental empty vector (EV) and treatment with SUMOylation inhibitors ML792 or TAK981 (5 μM), the MDM2 inhibitor Nutlin3A (5 μM) or control (DMSO 0.05%) for the last 24 h. Normality was assessed using the Shapiro-Wilk test. A one-way ANOVA statistical analysis followed by Tukey’s multiple comparison test was performed to compare transfection conditions within the same treatment. ∗ = *p* < 0.05, ∗∗ = *p* < 0.01, ∗∗∗ = *p* < 0.001, ∗∗∗∗ = *p* < 0.0001. Representative experiments from N = 3 independent experiments are shown. *B*, *D*, and *H*, protein expression was assessed by immunoblotting in the same extracts as in (*A*, *C*, and *G*). *E*, sequences of the 9 ZNF mutants generated *F*, immunoblot analysis of whole cell lysates HA-p53, HA-ZNF-p53, and the nine ZNF-fusion mutants following expression in HEK293 cells for 48 h. Cells were then lysed, and proteins were separated on a 7.5% SDS-PAGE, transferred to a membrane using a Trans-Blot Turbo system and immunoblots were performed using anti-HA antibody. The same amounts of transfected proteins were loaded on each gels. Representative immunoblots from two independent experiments (N = 2) are shown. DMSO, dimethyl sulfoxide.

p53 regulates the expression of a large number of genes, notably p21 whose upregulation leads to cell cycle arrest (55). To test whether ZNF-induced SUMOylation modulates p53 transcription activity, we cotransfected our constructs with the pG13-luc vector (56), a luciferase reporter containing p53-binding sequences of the p21 gene regulatory regions in HEK293 cells. In these assays, the expression of HA-ZNF-p53 resulted in a 3-fold reduction in transcriptional activity compared to the expression of HA-p53 (Fig. 6*A*), despite similar protein expression levels of HA-p53 and HA-ZNF-p53 (Fig. 6*B*). Low luciferase activity in the empty vector condition can be explained by the fact that the HEK293 cells used in this study harbor a R280S mutation of *TP53*. In the crystal structure of p53 bound to DNA (57), arginine 280 directly contacts DNA, establishing key interaction with an invariant guanine in the p53 consensus binding site and even a conservative mutation of this residue to a lysine residue leads to the loss of DNA binding (58).

MDM2 is a direct interactor and corepressor of p53 activity that can trigger p53 proteasomal degradation through its E3 ubiquitin ligase activity. As SUMOylation of MDM2 was previously shown to affect p53 abundance (59, 60), our ZNF fragment could affect the interaction and degradation by MDM2. We repeated our assay with cotreatment by Nutlin 3A (5 μM), an MDM2-p53 interaction inhibitor. Treatment with Nutlin 3A did not noticeably alter p53 abundance and transcriptional activity (Figs. 6*A* and S11*A*). We also noted similar abundance between endogenous p53 and transfected p53 (Fig. S11*A*). In contrast, treatment of cells with SUMO E1 inhibitors ML792 or TAK-981 led to the recovery of HA-ZNF-p53 transcriptional activity to levels comparable to those of HA-p53 (Fig. 6, *A* and *C*) without affecting the abundance of either fusion proteins or endogenous p53 (Fig. S11*B*). Combined treatment with Nutlin 3A and the SUMO E1 inhibitors did not further impact activity. Finally, fusion mutant HA-ZNF^AAAA^-p53 behaved in a manner similar to HA-ZNF-p53 (Fig. S11*B*). Overall, these results suggest that the ZNF fusion represses the transcription activity of p53 in a SUMOylation-dependent manner.

To assess whether the inhibitory effect of HA-ZNF^AAAA^-p53 on transcription activity assays may be due to the lack of abrogation of SUMOylation led us to investigate the impact of other mutations in ZNF. We generated eight additional HA-ZNF-p53 constructs with mutations in the ZNF tag (Fig. 6*E*). As these constructs expressed at different levels, we adjusted the amount of protein loaded on gels to normalize the p53 signal on the immunoblots (Fig. 6*F*). Short exposure revealed clear reduction of SUMOylation for all ZNF mutants compared to HA-ZNF-p53 (Fig. 6*F*). Residual SUMOylation was detected for the SIM1 AAAA mutant, but also for R40A and R40E, two mutations in the PLRP motif of ZNF that were previously reported to be essential for ZNF activity (28). Longer exposure showed that other ZNF mutants were still able to promote residual SUMOylation, including SIM2 AAAA where amino acids in the second SIM are replaced with alanine residues, and a mutant where two serine residues C terminal to SIM2 are replaced to alanine residues, thereby eliminating two putative phosphorylation sites. Notably, we did not detect any SUMOylation for HA-p53, the double SIM mutant (2 SIMs AAAA), the combined phosphorylation and SIM1 AAAA mutant, the SIM1 mutant where residues were changed to hydrophile residues (SIM1 NQSD), and a variant of ZNF where residues were scrambled. Luciferase assays performed with the non-SUMOylated HA-ZNF^2SIMsAAAA^-p53 construct indicated increased activity of this construct compared to HA-ZNF-p53 despite similar expression levels (Fig. 6, *G* and *H*). Treatments with TAK981 reestablished transcription activity of HA-ZNF-p53 to levels comparable to that of the mutant. Overall, these results indicate that altering several molecular determinants of ZNF strongly reduces its activity and that mutation of both SIMs appears required to abrogate residual SUMOylation activity.

### The ZNF tag increases SUMOylation of HSF1 and DNMT3A in transfected cells

**Figure 7:**
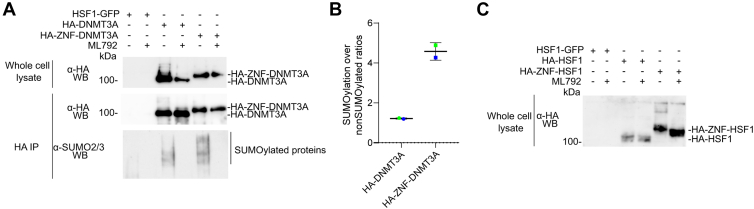
**Fusion of ZNF to DNMT3A and HSF1 increases their SUMOylation *in cellula.****A*, *C*, immunoblot analysis of *A*, whole cell lysate or immunoprecipitated HSF1-GFP, HA-DNMT3A, or HA-ZNF-DNMT3A or *C*, whole cell lysate of HEK293 cells transfected with HSF1-GFP, HA-HSF1, or HA-ZNF-HSF1 (*C*) following 48 h expression in HEK293 cells. Cell were lysed, then immunoprecipitation assays were performed overnight at 4 °C using anti-HA antibodies, followed by washes and elution in Laemmli buffer. Proteins were separated on a 7.5% SDS-PAGE. Immunoblots were performed using anti-HA antibodies on whole cell lysates and anti-HA or anti-SUMO2/3 antibodies on immunoprecipitated proteins. Representative immunoblots from two independent experiments (N = 2) are shown. *B*, quantification of SUMOylation levels was performed by measuring the band intensities for each condition of immunoprecipitated proteins in (*A*) and is presented relative to the control treatment. DNMT3A, DNA methyltransferase 3A; HSF1, heat shock factor 1.

Given that ZNF effectively enhances p53 SUMOylation, we investigated whether our approach could be extended to other proteins, particularly DNA binding proteins. On the one hand, we fused ZNF to DNMT3A, a protein known to influence gene expression through the regulation of DNA methylation (61) and a target for SUMOylation (62). Following transfection of HA-DNMT3A and HA-ZNF-DNMT3A, we immunoprecipitated the proteins using an anti-HA antibody (Fig. 7*A*). Immunoblotting proteins using an anti-SUMO2/3 antibody following HA-immunoprecipitation revealed that, while HA-DNMT3A undergoes basal SUMOylation, fusion of ZNF to the protein further increased the SUMOylation by ∼4.5 folds (Fig. 7*B*). For both proteins, treatments using ML792 inhibited DNMT3A’s SUMOylation. Interestingly, only the unmodified protein bands were detected in whole cell lysate, suggesting that only a small portion of the protein pool is modified. On the other hand, we fused ZNF to the HSF1, a transcription factor involved in heat stress response (63) that also undergoes SUMOylation (64). Anti-HA immunoblot of the whole cell lysates of transfected HEK293 cells showed that only HA-ZNF-HSF1 underwent detectable mono-SUMOylation, which was inhibited by treatment with ML792 (Fig. 7*C*). Overall, these results indicate that the ZNF tag can be used to SUMOylate proteins other than p53, showcasing its versatility.

## Discussion

This research focused on developing a novel strategy to promote targeted SUMOylation of a protein of interest. Based on the robust activity of the ZNF451 SUMO E3 module, we reasoned that its fusion to a target protein would result in increased SUMOylation, primarily *via* SUMO2, the preferred paralog used by ZNF451. The small size of the ZNF tag also minimizes possible steric effects on the fused substrate. As a proof of principle for the use of ZNF as a SUMOylation-inducing strategy, we used p53 as a target and monitored changes in its SUMOylation levels and transcriptional activation properties. The resulting p53 fusion protein was preferentially modified with SUMO2 compared to SUMO1, both *in vitro* and in transfected cells. Mass spectrometry experiments revealed that the canonical SUMOylation site of p53, K386, is also the main residue modified by ZNF in a cellular context, suggesting that ZNF does not strongly alter site specificity but rather enhance the efficacy of SUMOylation. Mutating the residues of the first SIM of ZNF451 to alanine residues reduced ZNF activity in *in vitro* and transfected cells. Under our transfection conditions, enhanced SUMOylation of p53 did not lead to significant increases in protein degradation but strongly affected p53 transcriptional activity in a p53 luciferase reporter assay. Finally, fusing ZNF to two additional proteins, HSF1 and DNMT3A, resulted in an augmentation of their SUMOylation, therefore showcasing that this strategy could be used for different proteins. Together, these results suggest that the fusion of proteins of interest with ZNF could be leveraged to promote their SUMOylation and regulate protein activity.

Overexpression of SUMOylation machinery components (SUMOs, E1, E2) can lead to an increased SUMOylation of a target protein but also a global SUMOylation increase. Among more targeted strategies used in the past to study protein SUMOylation and its impacts on targeted substrates (41, 46, 47, 48), fusion with SUMO paralogs has been used to replicate the effect of SUMOylation to a certain extent (65). Another strategy has relied on the fusion of an E2 to p53 (46). This approach, however, promotes SUMOylation by all SUMO types and could lead to increased global SUMOylation due to the overexpression of the unique SUMOylation E2 enzyme. In contrast, our approach does not alter concentrations of essential components of the SUMO machinery. The small size of the ZNF tag further limits the steric impact on the conformation of the targeted protein. In our proof-of-principle study, we showed that this approach maintains selectivity toward the main SUMOylation site of p53, as lysine 386 was the only modified residue detected by mass spectrometry in transfected cells (Fig. 4*E*). *In vitro*, mutation of lysine 386 reduced the kinetics of mono-SUMOylation (Figs. 3, S7 and S8). Other lysine residues were also modified *in vitro*, likely due to the efficacy of the SUMOylation reaction performed with purified enzymes. Of note, SUMOylation of the endogenous p53 protein at K320 has also been detected by mass spectrometry (66).

Contrary to what was observed with the E2-p53 fusions (46), we did not detect SUMO1-modified HA-ZNF-p53 in our assays in transfected cells (Fig. 4*C*), although our *in vitro* assays showed a modest yet consistent increase in SUMO1 modification when fusing ZNF to p53 (Fig. 2, *A* and *C*). This selectivity is consistent with the more robust SUMO E3 ligase activity of ZNF with SUMO2/3 both *in vitro* (27, 28) or *in vivo* (33), the higher concentrations of SUMO2/3 compared to SUMO1 in cells (24), and the lower efficacy of the SUMOylation reaction in transfected cells compared to *in vitro*.

A PLRP motif separates the two SIMs of ZNF451, and mutational analyses confirmed that both the integrity of the SIMs and that of the PLRP motif are essential to the full SUMO E3 activity of ZNF451 *in vitro* (27, 28). Although ZNF^AAAA^ SUMOylation activity was indeed minimal in *in vitro* experiments, HA-ZNF^AAAA^-p53 SUMOylation was readily detected in transfected HEK293 cells. Interestingly, in cells, several other mutations altering the molecular determinants of the ZNF tag reduced its activity relative to wild-type ZNF, without fully eliminating it, unless both SIM motifs were mutated (Fig. 6*F*). This suggests that additional SUMOylation mechanisms could also operate in cells. Notably, SUMO E3 ligases could be recruited by either SIM of ZNF. Recent work indeed suggests that the SUMOylation of many proteins is dependent on SUMO-SIM noncovalent interaction, perhaps through the recruitment of endogenous SUMO E3 ligases (67). Alternatively, the ZNF tag could target the attached protein to an environment more prone to SUMOylation, such as promyelocytic leukemia nuclear bodies (42). It is thus tempting to speculate that both the ZNF SUMO E3 ligase activity and its capacity to recruit or colocalize with other SUMO E3 ligase could contribute to the increased SUMOylation of p53. In addition, our present data do not exclude the possibility that ZNF promotes the SUMOylation of p53 interactors, in addition to p53 itself. Since we demonstrated that ZNF proximity can increase SUMOylation of a substrate, it is possible that protein brought into proximity of ZNF through interaction with p53 would also undergo increased SUMOylation.

Since the ZNF tag promotes modification of lysine residues, SUMOylation is predicted to crosstalk with other PTMs that target SUMOylation sites, such as lysine acetylation or ubiquitination. K386 is a well-characterized site of ubiquitination by MDM2, p53’s main negative regulator (69, 70). However, blocking MDM2 interaction with p53 did not lead to a significant accumulation of endogenous p53, HA-p53, or HA-ZNF-p53 under the conditions or our assays (Fig. 6). SUMOylation could play a protective role against proteasome degradation of some target proteins, acting as a ubiquitin competitor or a signal leading to inhibition of ubiquitination, as it was observed for instance with PKCδ (71). Mass spectrometry experiments on whole cell lysate also revealed that phosphorylation of serine 392 of p53 was detected together with lysine 386 SUMOylation in the HA-ZNF-p53 fusion (Fig. 4*E*). The interplay between phosphorylation and SUMOylation has previously been reported, specifically in phosphorylation-dependent SUMOylation motif (72). One notable example is HSF1, for which phosphorylation is important for SUMOylation and efficient heat stress response (73). In the case of ZNF fusion-induced SUMOylation for p53, phosphorylation of serine 392 may facilitate SUMOylation at residue 386. Alternatively, SUMOylation might induce phosphorylation of proximal serine residue (74), which was reported to promote apoptosis (75). Whether ZNF could induce other PTMs through SUMOylation remains to be established.

Although SUMOylation can protect against ubiquitination and degradation when a ubiquitination site becomes SUMOylated, poly/multi-SUMOylation with SUMO2/3 is sometimes associated with ubiquitination by the STUbLs RNF4 or RNF111 and protein degradation (76). In our experiments, we did not observe evidence for the ubiquitination or degradation of poly/multi-SUMOylated HA-ZNF-p53 (Fig. 5, *D* and *E*). This may be due to an insufficient level of poly-SUMOylation or of STUbL proteins in our model cell lines to modify our exogenous fusion protein effectively. It should be noted that poly-SUMOylation has been shown to modulate the transcriptional activity of other transcription factors rather than promoting their degradation. Nrf2, a transcription factor implicated in oxidative stress response, is recognized by the STUbL RNF111 when poly-SUMOylated. This leads to its ubiquitination but stabilizes rather than degrades Nrf2 (77). Although we expected HA-ZNF-p53 to undergo increased multi/poly-SUMOylated following MG132 treatment, the opposite effect was observed. This could be due to increased ubiquitination competing with SUMOylation. Another explanation is that anti-SUMO2/3 immunoblots on whole cell lysates indicated a depletion of the free SUMO2/3 pool upon proteasome inhibition and this behavior was exacerbated for cells transfected with HA-ZNF-p53. It is thus tempting to speculate that ZNF activity may be dependent on the size of the free SUMO2/3 pool, especially given the requirement of two SUMO on the E2 for ZNF association and activity.

The fusion of ZNF to p53 appeared to promote poly/multi-SUMOylation of the entire pool of p53 during *in vitro* assays (Fig. 1*B*), but only a fraction of the total p53 pool underwent SUMOylation in cells (Figs. 4, *A*, *B* and 5, *A B*). This is particularly apparent when comparing the SUMOylation levels of HA-ZNF-p53 to those of HA-p53-SUMO2 (Fig. 4*B*). It was previously reported that less than 5% of endogenous p53 is SUMOylated under normal conditions (78), and that modification of the p53 tetramer is substoichiometric (79). However, it has been suggested that only partial protein SUMOylation is required to observe major effects on transcription activity (80, 81). Our data indicate that, even if only a portion of the p53 pool underwent SUMOylation, the impact on its transcriptional activity can still be significant (Fig. 6, *A*, *C* and *G*).

In our assays, forced SUMOylation of p53 by endogenous levels of SUMO proteins and SUMO E1 and E2 enzymes suppressed its transcriptional activity (Fig. 6). In the literature, the exact impact of SUMOylation on p53 activity appears variable according to experimental conditions. Activation of p53 by SUMOylation with overexpressed SUMO1 was reported in U2OS cells using the same reporter vector as in our study (49). Overexpression of SUMO2/3 in HEK293 cells also resulted in higher transcriptional activity when using a pRGC-luc, a reporter plasmid based on the ribosomal RNA gene cluster promoter (41). Similarly, repression of SENP1, a SUMO protease, resulted in increased p53 transcriptional activity for some known target genes, such as p21 (82). On the other hand, rapamycin-induced interaction between p53 and SUMO2/3 led to lower transcriptional activity of p53 (83), consistent with our results. These differential effects could also be due to a global increase in SUMOylation *versus* targeted modification of a specific protein. Importantly, our results indicate that the activity of all SUMOylated p53 constructs was restored to levels comparable to those of HA-p53 upon SUMO E1 inhibition, indicating that the repressive effects of the fusion tags are SUMOylation-dependent.

Several reasons could explain the decrease in p53 transcriptional activity upon forced SUMOylation. SUMOylation of p53 was reported to block acetylation at C-terminal lysine residues, inhibiting DNA binding (79). Reduced activity of SUMOylated p53 may also result from changes in subcellular localization. SUMOylation of p53 using a rapamycin-induced interaction to a chemically fused SUMO was reported to cause CRM1-mediated export from the nucleus to the cytoplasm (83). Also, distribution within the nucleus could be altered by SUMOylation. For example, p53 was shown to transit to promyelocytic leukemia nuclear bodies (42), but the exact consequences of this localization on its activity remain to be determined. Additional experiments will be needed to explore whether the ZNF tag influences p53 subcellular localization in the cytoplasm. Finally, SUMOylated p53 could recruit common corepressors such as CoREST1 (84), NCoR1 (85), or some HDACs (85, 86), which are known to interact directly with SUMO through their SIMs (22), leading to the lower observed transcription activity. Furthermore, the activity of corepressor complexes recruited in a SUMO-dependent manner can be itself increased by SUMOylation, while coactivators can be inhibited (87, 88).

Overall, our results indicate that the ZNF tag can successfully increase SUMOylation when fused to a substrate. The small size of the ZNF tag will facilitate further development for biotechnological or therapeutic applications whereas the capacity of ZNF-mediated SUMOylation to modulate the activity of transcription factors may facilitate transcriptional reprograming in diseases such as cancer.

## Experimental procedures

### Reagents

HEK293 cells were cultured in Dulbecco’s Modified Eagle Medium (DMEM, Wisent, Cat. No. 319-016 CL) containing 10% fetal bovine serum (Wisent, Cat. No. 090-150) in 5% CO_2_-containing humidified air at 37 °C. Cells were routinely tested for *mycoplasma* contamination. Polyethylenimine (PEI) was purchased from Polysciences (Cat. No. 23966-1). Protease inhibitor cocktail was from Selleckchem (Cat. No. B14002). N-Ethylmaleimide (NEM, Cat. No. 04259-5G) and Cycloheximide (CHX, Cat. No. C7698) were purchased from Sigma-Aldrich. Sulfamic acid, [(1R,2S,4R)-4-[[5-[[1-[(3-bromophenyl)methyl]-1H-pyrazol-3-yl]carbonyl]-4-pyrimidinyl]amino]-2-hydroxycyclopentyl]methyl ester (ML792) was from Cayman Chemical (Cat. No. 36820). [(1*R*,2*S*,4*R*)-4-[[5-[4-[(1*R*)-7-chloro-1,2,3,4-tetrahydroisoquinolin-1-yl]-5-methylthiophene-2-carbonyl]pyrimidin-4-yl]amino]-2-hydroxycyclopentyl]methyl sulfamate (TAK981) was from MedChemexpress (Cat. No. HY-111789). Carbobenzoxy-L-leucyl-L-leucyl-L-leucinal (MG132) was from Ubiquitin-Proteasome Biotechnologies LLC (UBPBio) (Cat. No. F1101).

Only commercial antibodies validated by the manufacturer were used for this study. Precisely, the following antibodies were used: Rabbit anti-SUMO2/3 (1:750; Sigma-Aldrich, Cat. No. S9571), Mouse anti-SUMO1 (1:1500; UBPBio, Cat. No. Y3111), Mouse anti-HA (1:1000; BioLegend, Cat. No. 16B12), Rabbit anti-HA (1:750; BioLegend Cat. No. 902302), Mouse anti-ubiquitin – P4D1 (1:750; Santa Cruz, Cat. No. sc-8017), Mouse anti-p53 (1:1000; Santa Cruz, Cat. No. sc-98), HRP-conjugated Rabbit anti-Mouse (1:10,000; Cell Signaling, Cat. No. 7076S), HRP-conjugated Donkey anti-rabbit (1:10,000; Cell Signaling, Cat. No. 7074S).

### Constructions

SUMO2^15–93^ in which all lysine residues were mutated to arginine (SUMO2 K0), was synthesized (Integrated DNA Technologies). SUMO1^1–97^, SUMO2^1–93^, and SUMO2 K0 were cloned by Gibson assembly into the BamHI site of a modified pRSFDuet plasmid where the thrombin site was replaced by a site for the tobacco etch virus (TEV) protease. A codon-optimized version of human Ubc9, herein called E2, was synthesized (Integrated DNA Technologies) and cloned into the NdeI-XhoI site of pRSFDuet to produce a wild-type untagged protein. The pET28b/ULP1^403–621^, pET11c/SAE1 and pET28b/SAE2^1-550^ vectors were obtained from Christopher D. Lima. ZNF (ZNF451^24–55^) was synthesized (Integrated DNA Technologies), while pcDNA3 p53 WT was a gift from David Meek (Addgene plasmid No. 69003; http://n2t.net/addgene:69003; RRID:Addgene_69003). HA-p53, ZNF, and HA-ZNF-p53 (WT, and K386R) were cloned by Gibson assembly into the BamHI site of a pTRX vector for bacterial protein expression and into the HindIII-XhoI site of pcDNA3.1 (+) (Invitrogen) for expression in human cells. HA-HSF1, HA-ZNF-HSF1, HSF1-GFP, HA-DNMT3A, and HA-ZNF-DNMT3A were cloned into the HindIII-XhoI site of pcDNA3.1 (+) (Invitrogen) for expression in human cells. The pTRX vector is a homemade pRSFDuet-based plasmid that allows the production of proteins as His6-thioredoxin fusions with a TEV cleavage site immediately following the thioredoxin sequence. HA-p53-SUMO1^1–93^ and HA-p53-SUMO2^1–89^ were cloned by Gibson assembly into the HindIII-XhoI site of pcDNA3.1 (+) (Invitrogen). HSF1-GFPN3 was a gift from Stuart Calderwood (Addgene plasmid No. 32538; http://n2t.net/addgene:32538; RRID:Addgene_32538). PG13-luc containing consensus p53 binding sites was a gift from Bert Vogelstein (Addgene plasmid No. 16442; http://n2t.net/addgene:16442; RRID:Addgene_16442). Except for the scrambled version of ZNF, all the mutants of HA-ZNF-p53 (R40A, R40E, SIM1 AAAA, SIM2 AAAA, 2SIMs AAAA, phospho AA, phospho AA + SIM1 AAAA, SIM1 NQSD) were generated by site-directed mutagenesis. The Q88R mutants of SUMO2 and SUMO2 K0 were also obtained by site-directed mutagenesis. Constructs and mutations were confirmed by Sanger sequencing.

### Protein expression in bacteria and purification

Bacterial expression vectors were transformed by heat shock in BL21 DE3 Gold Codon Plus RIL, which were then grown overnight at 37 °C in Super Broth (32 g/L tryptone, 20 g/L yeast extract, and 5 g/L NaCl) containing the appropriate antibiotics (34 μg/ml chloramphenicol, 50 μg/ml kanamycin, or 100 μg/ml ampicillin). Precultures were then diluted 1:3 with chilled Super Broth media containing antibiotics and 0.3 M IPTG and incubated for 24 h at 18 °C. Cells were pelleted by centrifugation at 6000 *g* for 40 min. Pellets were resuspended in sucrose buffer (20% sucrose, 50 mM Tris–HCl pH 8.0) using 2 ml per gram of pellet. Resuspended bacteria were snap-cooled in liquid nitrogen and kept at −20 °C until needed.

For SAE1/SAE2^1-550^, SUMO1, SUMO2, SUMO2 K0, ULP1^403–621^, HA-p53, and HA-ZNF-p53 (WT and mutants), bacteria were thawed, and the solution composition was adjusted to 500 mM NaCl, 20 mM imidazole, and 5 mM β-mercaptoethanol. The solution composition of E2-expressing bacteria was adjusted to 20 mM NaCl and 5 mM β-mercaptoethanol. In all cases, bacteria were sonicated (Branson Sonifier 450) for 3 cycles of 2 min, at a power of 200W and a 1:1 duty cycle, then centrifugated at 18,000 g for 25 min. Supernatants were used for gravity-flow purification. Precisely, E2 was purified using 1 ml of SP-resin (GE Healthcare) per liter of culture using 20 column volume (CV) of wash buffer (20 mM Tris–HCl pH 8.0, 20 mM NaCl, and 5 mM β-mercaptoethanol) and 10 CV of elution buffer (20 mM Tris–HCl pH 8.0, 500 mM NaCl, and 5 mM β-mercaptoethanol). Other proteins were purified using 2 ml of NiNTA (Qiagen) resin pre-equilibrated in NiNTA wash buffer (20 mM Tris-HCl pH 8.0, 500 mM NaCl, 20 mM imidazole, and 5 mM β-mercaptoethanol) per liter of culture. Washes were performed using 15 CV of NiNTA wash buffer and protein was eluted with 5 CV of NiNTA elution buffer (20 mM Tris-HCl pH 8.0, 500 mM NaCl, 500 mM imidazole, 5 mM β-mercaptoethanol). In all cases, protein concentration in the eluted fractions was determined using absorbance at 280 nm, and the six most concentrated 1 ml fractions were combined for further purification.

SAE1/SAE2, SUMO1, SUMO2, ULP1, HA-p53, and every fusion protein were cleaved overnight at 4 °C using 1 μM TEV protease. His tag (for SUMO1, SUMO2, and SUMO2 K0) or His-thioredoxin tag (for other constructions) removal was validated through migration on SDS-PAGE before subsequent purifications. All proteins were then further purified using size-exclusion chromatography in gel filtration buffer (20 mM Tris–HCl pH 8.0, 150 mM NaCl, and 5 mM β-mercaptoethanol) on an AKTA Pure FPLC (Cytiva). Proteins under 50 kDa were purified on a S75 16/60 column (Cytiva) while proteins over 50 kDa were purified on a S200 16/60 column (Cytiva). Protein purity was confirmed using SDS-PAGE, and protein concentration in the eluted fractions was determined using absorbance at 280 nm. Purified proteins were concentrated, aliquoted, flash-cooled in liquid nitrogen, and stored at −80 °C until needed.

### *In vitro* SUMOylation assays

SUMOylation reactions were performed in a buffer containing 20 mM Hepes pH 7.5, 125 mM NaCl, 2 mM MgCl_2_, 0.1% Tween, and 3 mM DTT. All reactions used the following protein concentrations: 100 nM E1, 100 nM E2, and 50 μM SUMO. For control reactions without E3, 1 μM of HA-p53 was used. For reactions with an E3 added in *trans*, 1 μM of ZNF was used. For all the reactions using fusion proteins, 1 μM of HA-ZNF-p53 or its mutated version were used. Reactions were all performed at 37 °C and were started with the addition of ATP at a final concentration of 2 mM. Reactions were stopped at 0, 10, 20, 40, and 80 min by adding Laemmli buffer. For ULP1-treated samples, after the last time point, 1 μM ULP1 was added to the reactions for 10 min, then Laemmli was added to stop the reaction. Samples were loaded on a 12% SDS-PAGE gel. Migrations were done at 180V for 55 min. Gels were stained using Coomassie Blue, and images were acquired using a ChemiDoc MP (Bio-Rad). Quantifications were performed using ImageLab. The relative abundance of SUMOylated and non-SUMOylated substrate was determined by measuring the band intensity corresponding to these different forms. Quantification of diSUMO was performed by measuring the band intensity at each time point. Experiments were conducted in technical triplicates using single-use protein aliquots.

### Transfection of HEK293 cells

For immunoprecipitation assays, cells were transfected while in suspension using linear PEI (1:3 w/w ratio of plasmid to PEI) before being plated in DMEM medium in 6-well cell plates (Sarstedt; 1,000,000 cells/well). For 48 h assays, 1 μg of plasmid was transfected, while amounts of cells, PEI and plasmid were doubled for 24 h transfection. For experiments using HSF1, quantities of plasmid and PEI were also doubled to compensate for lower HSF1 expression. Cells were treated with ML792 (5 μM) for 4 h or with vehicle (DMSO 0.01%). For protein synthesis and degradation studies, cells were treated with CHX (30 μg/ml) for 30 min before treatments with MG132 (20 μM) or vehicle (DMSO, final concentration of 0.02%) for 4 h.

For luciferase assays, cells were similarly transfected with linear PEI (1:3 w/w ratio of 1 μg DNA per 3 μg PEI) after plating in 6-well plates in DMEM medium supplemented with 10% fetal bovine serum (300,000 cells/well). Transfection was performed 6 h after cell plating with the pG13-luc reporter (1 μg), HA-p53, HA-ZNF-p53 or HA-ZNF^AAAA^-p53 (0.5 μg), as well as an eGFP expression vector (0.01 μg) to normalize transfection efficiency. Eighteen hours after transfection, cells were trypsinized, resuspended in DMEM medium, seeded into 96-well solid white flat bottom assay plates at 30,000 cells per well and left to adhere for 6 h. Cells were then treated with a SUMO E1 inhibitor (TAK981 or ML792) and/or Nutlin 3A for 24 h at 5 μM final concentration for each inhibitor or with vehicle (DMSO 0.05% EtOH 0.5% final concentration).

### Immunoprecipitation assays

Cells were washed twice with PBS and incubated 5 min on ice with 1 ml of ice-cold Lysis Buffer (20 mM Tris–HCl pH 7.5, 150 NaCl, 1% Triton X-100, 5 mM EDTA, 0.1% SDS, 1× protease inhibitor cocktail) containing NEM (20 mM) to limit protein deSUMOylation by SUMO proteases. The solution was then agitated at 20 rpm for 30 min at 4 °C. Lysates were centrifuged at 21,000 *g* for 15 min at 4 °C and supernatants were kept for immunoprecipitations. One microgram of HA antibody and 10 μl of Protein A/G plus-agarose resin (Santa Cruz, Cat. No. sc-2003) were added to the supernatants before overnight incubation at 4 °C in a rotating mixer. The following day, the resin was washed three times using lysis buffer without inhibitors. After the last wash, Laemmli buffer 2× (40 μl) was added to the resin, and the solution was incubated at 95 °C for 3 min before loading on SDS-polyacrylamide gels.

### Western blotting

Samples were loaded on 7.5% SDS-polyacrylamide 1 mm gels containing 0.5% 2,2,2-trichloroethanol and migrated at 180V for 55 min. Gels were imaged with a ChemiDoc MP using the stain-free gel protocol of ImageLab and 45 s of activation. Proteins were then transferred to a 0.45 μm polyvinylidene fluoride membrane (Millipore) using the wet transfer method and Towbin buffer with SDS (25 mM Tris–HCl, 192 mM glycine, 20% methanol, and 0.3% SDS). When specified, proteins were instead transferred using Trans-Blot Turbo system (Bio-Rad,) for 7 min, at 25V and 2.5A. Wet transfer was performed at 4 °C for 2 h at 400 mA. Blots were imaged with a ChemiDoc MP using the Stain-free blot protocol of ImageLab. Membranes were blocked using Tris-buffered saline with Tween 20 (TBST 1X, 20 mM Tris–HCl pH 7.5, 137 mM NaCl, 0.15% Tween 20 pH 7.5) with 5% w/v nonfat dry milk for 1 h. After three washes with TBST 1X, membranes were incubated with primary antibodies diluted in TBST+0.05% NaN_3_ for 1 h at room temperature. Following three TBST washes, membranes were incubated in a TBST solution containing the secondary antibody for 1 h. After three washes, Clarity Western ECL substrate (Bio-Rad, Cat. No. 1705061) was applied on the membrane and chemiluminescence was captured with a Chemidoc MP using ImageLab. The SUMOylated over non SUMOylated protein ratios were obtained by dividing the relative band intensities measured in anti-SUMO2/3 immunoblot by the relative band intensities measured in the HA immunoblots.

### Sample preparation for mass spectrometry analyses of *in vitro* SUMOylation reactions

SUMOylation assays were performed as mentioned before but using a buffer composed of 50 mM Tris–HCl pH 7.5, 5 mM MgCl_2_, and 3 mM DTT. The reactions were started by the addition of ATP at a final concentration of 5 mM. After 10 min, sample were incubated at 95 °C for 5 min and then snap-frozen in liquid nitrogen. Sixty microliters of 500 mM ammonium bicarbonate (ABC) was added to each sample to obtain a final concentration of 100 mM ABC. Trypsin (3.3 μg) was added to each sample, followed by overnight digestion at 37 °C in a thermomixer. The digested peptides were desalted using C18 StageTips (Cell Signaling Technology, Cat. No. 45943S) according to the manufacturer’s instructions. Desalted samples were dried completely in a SpeedVac.

### Sample preparation for mass spectrometry analyses of SUMOylation in tranfected cells

For detection of SUMOylated lysine residues in HEK293 cells stably expressing 6xHis-SUMO3-Q87R/Q88N was performed as previously described (17). Briefly, HEK293 SUMO3m cells were transfected with either HA-p53 or HA-ZNF-p53 vectors. Forty-eight hours post transfection, cells were collected and lysed in 50 mM Tris–HCl (pH 7.6), 1.5 mM MgCl_2_, 420 mM NaCl, 0.2 mM EDTA, and 25% glycerol, supplemented with 20 mM NEM, 1 × protease inhibitor cocktail, and 1 × phosphatase inhibitor cocktail (Sigma Aldrich, Cat. No. P0044). Protein concentrations were determined by Bradford assay. Immunoprecipitation of HA-tagged proteins was performed with 4 mg of total protein per condition as described above. Beads were washed three times with lysis buffer, and proteins were digested on-bead with 5 μg trypsin in 2 ml of 50 mM ABC at 37 °C overnight. The resulting peptide mixtures were dried directly using a SpeedVac.

### Analysis by liquid chromatography with tandem mass spectrometry (LC-MS/MS)

For *in vitro* SUMO samples, dried peptides were reconstituted in 12 μl of LC buffer A (4% formic acid in water), and 10 μl was injected. Peptide separation was performed on an IonOpticks Aurora Ultimate 25 cm × 75 μm ID, 1.7 μm C18 column using a Vanquish Neo HPLC system (Thermo Fisher Scientific). Peptides were resolved using a 5 to 35% linear gradient of LC buffer B (0.1% formic acid in acetonitrile) over 51 min at 0.3 μl/min and analyzed on an Orbitrap Exploris 480 mass spectrometer (Thermo Fisher Scientific) operated in positive ion mode. Full mass spectrometry survey scans were acquired at 120,000 resolution (m/z 200) across the m/z range 400 to 1800, with automatic gain control (AGC) target and maximum injection time set in standard mode. Data-dependent acquisition was performed in a 3 s cycle on the most intense precursors with a 1 Da isolation window. Precursor ions were fragmented by higher-energy collisional dissociation at a normalized collision energy of 30%, and tandem mass sectrometry (MS/MS) spectra were collected at 60,000 resolution (m/z 200) with a normalized AGC target of 100% and a maximum injection time of 123 ms.

For HEK293 samples, 10 μl of peptide solution was injected and separated on an IonOpticks Aurora Elite 15 cm × 75 μm ID, 1.7 μm C18 column using an Evosep One HPLC system (Evosep Biosystems). Peptides were eluted with 20 samples-per-day method and analyzed on an Orbitrap Ascend Tribrid mass spectrometer (Thermo Fisher Scientific) in positive ion mode. Full mass spectrometry survey scans were acquired at 120,000 resolution (m/z 200) across the m/z range 350 to 1200. Data-dependent acquisition was performed in a 3 s cycle with a 1.2 m/z isolation window. Precursor ions were fragmented by higher-energy collisional dissociation at a normalized collision energy of 28%, and MS/MS spectra were acquired at 22,500 resolution (m/z 200) with a normalized AGC target of 100% and maximum injection time of 43 ms.

### Processing of mass spectrometry data

Raw MS/MS data were processed with MaxQuant (version 2.2.0) using the UniProt/Swiss-Prot human protein database (release of November 13, 2021). Precursor mass tolerances were set to 20 ppm for the first search and 4.5 ppm for the main search, with a fragment mass tolerance of 7.5 ppm. Trypsin/P was specified as the digestion enzyme, allowing up to three missed cleavages. Carbamidomethylation of cysteine was set as a fixed modification. Variable modifications included methionine oxidation, N-terminal acetylation, and the SUMO2 remnant (+QQTGG) for *in vitro* SUMO samples, and methionine oxidation, asparagine/glutamine deamidation, serine/threonine/throsine phosphorylation, N-terminal acetylation, and the SUMO3 remnant (+NQTGG) for *in cellula* SUMO assays. A minimum peptide length of six amino acids was required, and a 1% false discovery rate was applied at both the peptide and protein levels. Only sites with a localization probability >0.75 were retained for downstream analyses.

### SUMOylation site prediction

PhosphoSite Plus v6.8.1, GPS-SUMO v2.0, and DeepSUMO were used for predictions of SUMOylation sites using p53 human sequence (P04637) (89, 90, 91, 92, 93).

### Luciferase activity assays

Cells were washed with PBS 1X, eGFP counts were acquired (excitation at 485 nm, emission at 538 nm and cut off at 530 nm) using a Flexstation II fluorescent plate reader. Cells were then assessed for luciferase activity in the presence of a luciferin lysis solution (30 mM Tricine, 1.61 mM (MgCO_3_)_4_•Mg(OH)_2_•5H_2_O, 4 mM MgSO_4_, 0.15 mM EDTA pH 7.0, 50 mM DTT, 0.81 mM coenzyme A, 0.7 mM D-luciferin, 0.8 mM ATP, 1% Brij58, 100 mM Trizma Acetate, 20 mM (CH_3_COO)_2_ Mg, and 2 mM EGTA in PBS 1 × ). Luminescence measurements were acquired after 10 min incubation at room temperature on a Spark Multimode Microplate Reader. Relative luciferase signals were obtained by normalization to eGFP expression, and each biological replicate was standardized to the control condition (HA-p53 overexpression without treatment).

## Data availability

The authors confirm that the data supporting the findings of this study are available within the article and its supporting information. The mass spectrometry proteomics data have been deposited to the ProteomeXchange Consortium (http://proteomecentral.proteomexchange.org) *via* the PRIDE partner repository with the dataset identifier PXD068083.

## Supporting information

This article contains supporting information.

## Conflict of interest

The authors declare that they have no conflicts of interest with the contents of this article.
