# Supplementary material for: A short SUMOylation tag modulates transcription factor activity
Source: J Biol Chem. 2025 Oct 8;301(11):110807. doi: 10.1016/j.jbc.2025.110807 (PMC12630355; doi:10.1016/j.jbc.2025.110807)
Supplement: Supporting Figures [file mmc1.pdf]

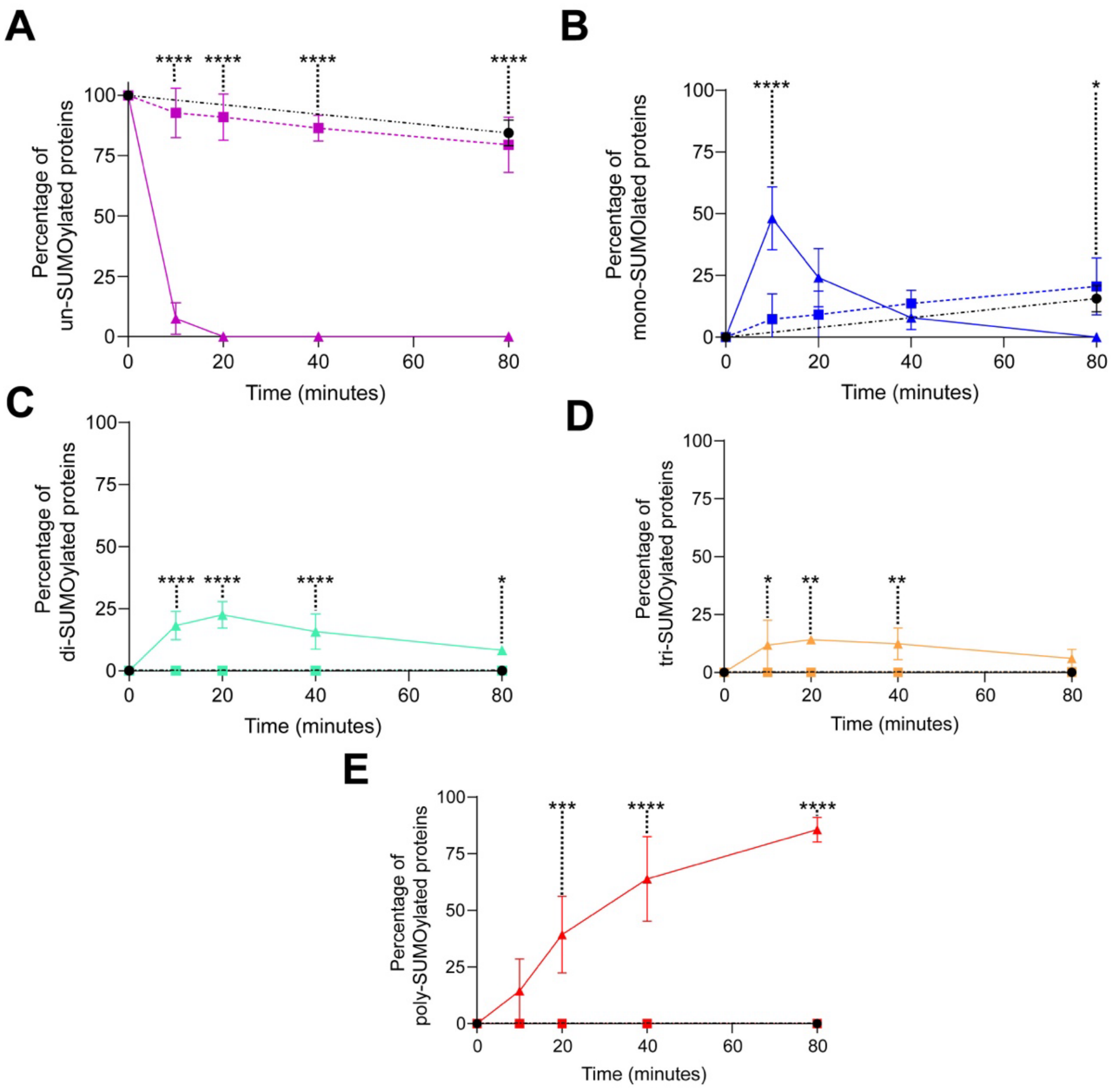

Figure S1: Quantification of SUMOylation associated to the HA-p53, HA-p53 + ZNF and HA-ZNF-p53 *in vitro* assays with SUMO2

Detailed quantifications from the *in vitro* assays presented in Figures 1B and 1C comparing SUMOylation levels of HA-p53, HA-p53 + ZNF, and HA-ZNF-p53. Relative abundance of (A) un-SUMOylated (B) mono-SUMOylated, (C) di-SUMOylated, (D) tri-SUMOylated, and (E) poly-SUMOylated proteins determined by quantification of band intensities for the conditions presented in Figure 1B. Colors identifying the different stages of SUMOylation are kept the same as Figure 1B-C. (●) refers to HA-p53, (■) to HA-p53 + ZNF, and (▲) to HA-ZNF-p53. A two-way ANOVA multiple comparisons statistical analysis, where the mean of HA-ZNF-p53 and HA-p53 + ZNF was performed to compare each stage protein modification (*=$p<0.05$, **=$p<0.01$, ***=$p<0.001$, ****=$p<0.0001$). HA-p53 (in black) was excluded from this statistical analysis, and only acts as a reference point.

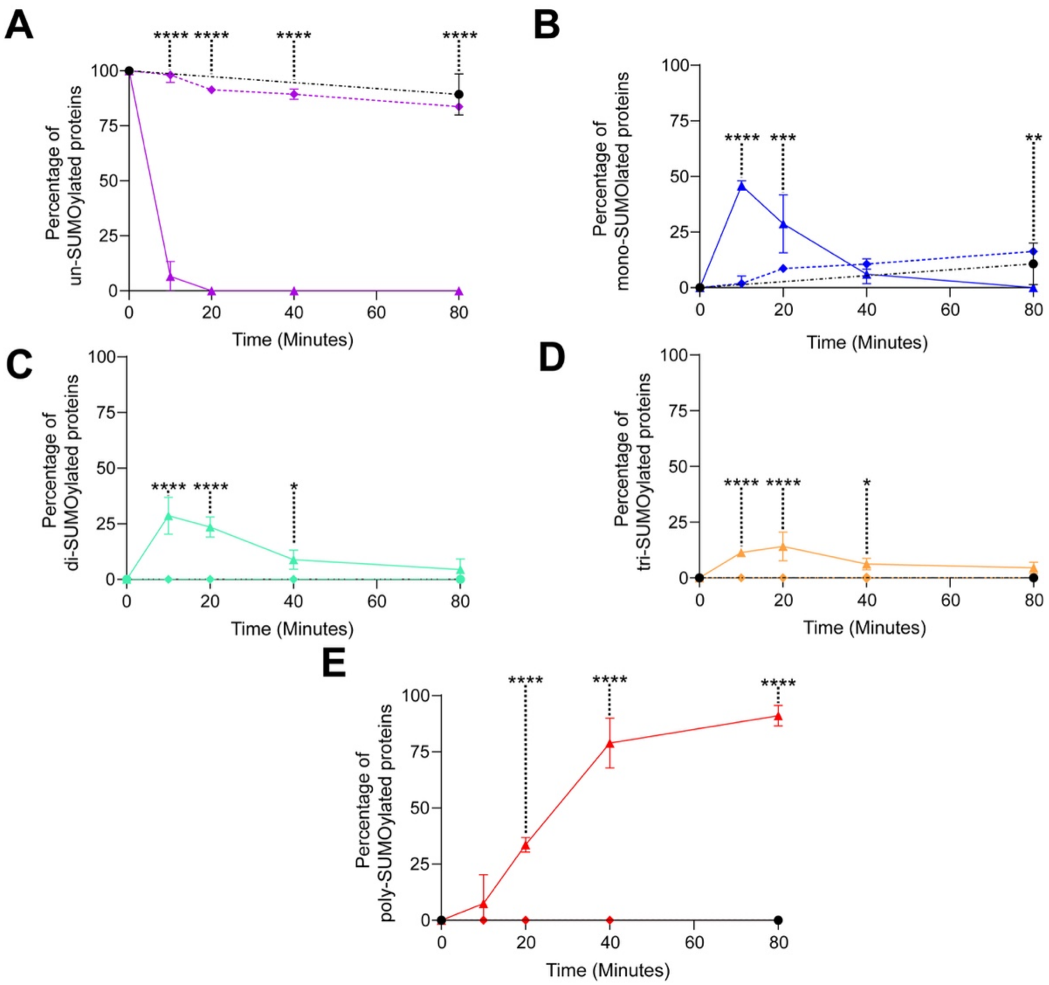

Figure S2: Quantification of SUMOylation associated to the HA-p53, HA-ZNF-p53, and HA-ZNF^{AAAA}-p53 *in vitro* assays with SUMO2

Detailed quantifications from the *in vitro* assays presented in Figures 1E and 1F comparing SUMOylation levels of HA-p53, HA-p53 + ZNF, and HA-ZNF-p53. Relative abundance of (A) un-SUMOylated (B) mono-SUMOylated, (C) di-SUMOylated, (D) tri-SUMOylated, and (E) poly-SUMOylated proteins determined by quantification of band intensities for the conditions presented in Figure 1E. Colors identifying the different stages of SUMOylation are kept the same as Figure 1F. (●) refers to HA-p53, (▲) to HA-ZNF-p53, and (♦) to HA-ZNF^{AAAA}-p53. A two-way ANOVA multiple comparisons statistical analysis, where the mean of HA-ZNF-p53 and HA-ZNF^{AAAA}-p53 was performed to compare each stage protein modification (*=$p<0.05$, **=$p<0.01$, ***=$p<0.001$, ****=$p<0.0001$). HA-p53 (in black) was excluded from this statistical analysis, and only acts as a reference point.

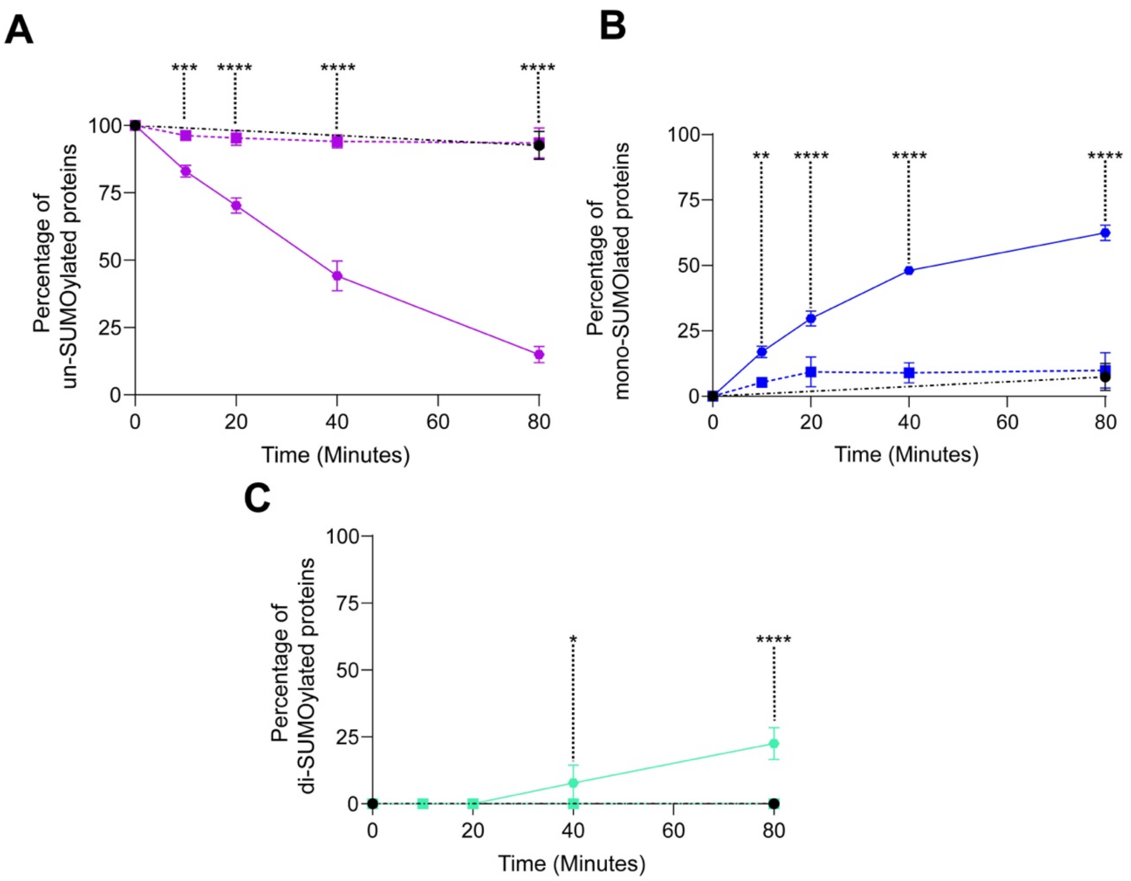

Figure S3: Quantification of SUMOylation associated to the HA-p53, HA-p53 + ZNF, and HA-ZNF-p53 *in vitro* assays with SUMO1

Detailed quantifications from the *in vitro* assays presented in Figures 2A and 2B comparing SUMOylation levels of HA-p53, HA-p53 + ZNF, and HA-ZNF-p53. Relative abundance of (A) un-SUMOylated (B) mono-SUMOylated, (C) di-SUMOylated, (D) tri-SUMOylated, and (E) poly-SUMOylated proteins determined by quantification of band intensities for the conditions presented in Figure 2A. Colors identifying the different stages of SUMOylation are kept the same as Figure 2B. (●) refers to HA-p53, (▲) to HA-p53 + ZNF, and (⬢) to HA-ZNF-p53. A two-way ANOVA multiple comparisons statistical analysis, where the mean of HA-ZNF-p53 and HA-p53 + ZNF was performed to compare each stage protein modification (*=$p<0.05$, **=$p<0.01$, ***=$p<0.001$, ****=$p<0.0001$). HA-p53 (in black) was excluded from this statistical analysis, and only acts as a reference point.

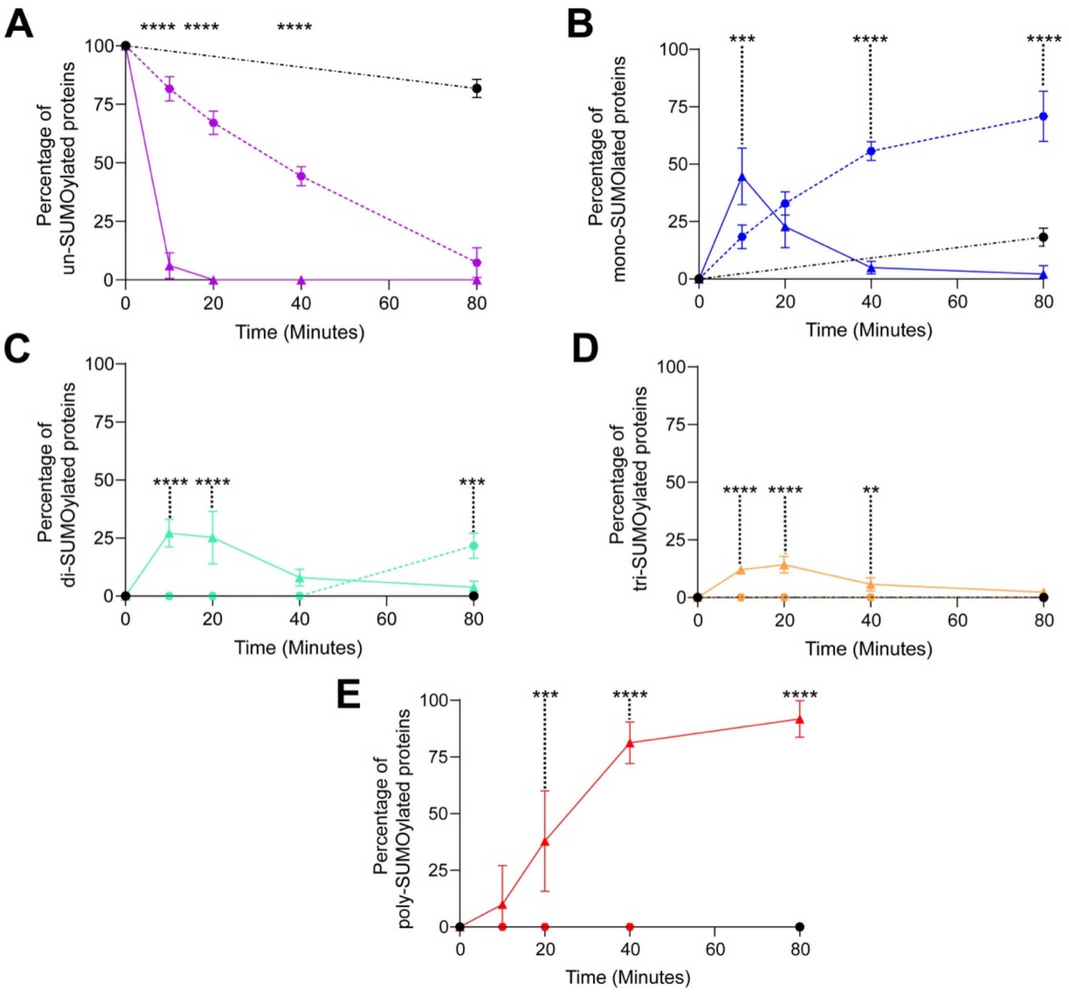

Figure S4: Quantification of SUMOylation associated to the HA-p53, and HA-ZNF-p53 *in vitro* assays with SUMO2 or SUMO1

Detailed quantifications from the *in vitro* assays presented in Figures 2C and 2D comparing SUMOylation levels of HA-p53, HA-p53 + ZNF, and HA-ZNF-p53. Relative abundance of (A) un-SUMOylated (B) mono-SUMOylated, (C) di-SUMOylated, (D) tri-SUMOylated, and (E) poly-SUMOylated proteins determined by quantification of band intensities for the conditions presented in Figure 2C. Colors identifying the different stages of SUMOylation are kept the same as Figure 2D. (●) refers to HA-p53, (▲) to HA-ZNF-p53 with SUMO2, and (⬣) to HA-ZNF-p53 with SUMO1. A two-way ANOVA multiple comparisons statistical analysis, where the mean of HA-ZNF-p53 with SUMO2 and with SUMO1 was performed to compare each stage protein modification (*=$p<0.05$, **=$p<0.01$, ***=$p<0.001$, ****=$p<0.0001$). HA-p53 (in black) was excluded from this statistical analysis, and only acts as a reference point.

A

B

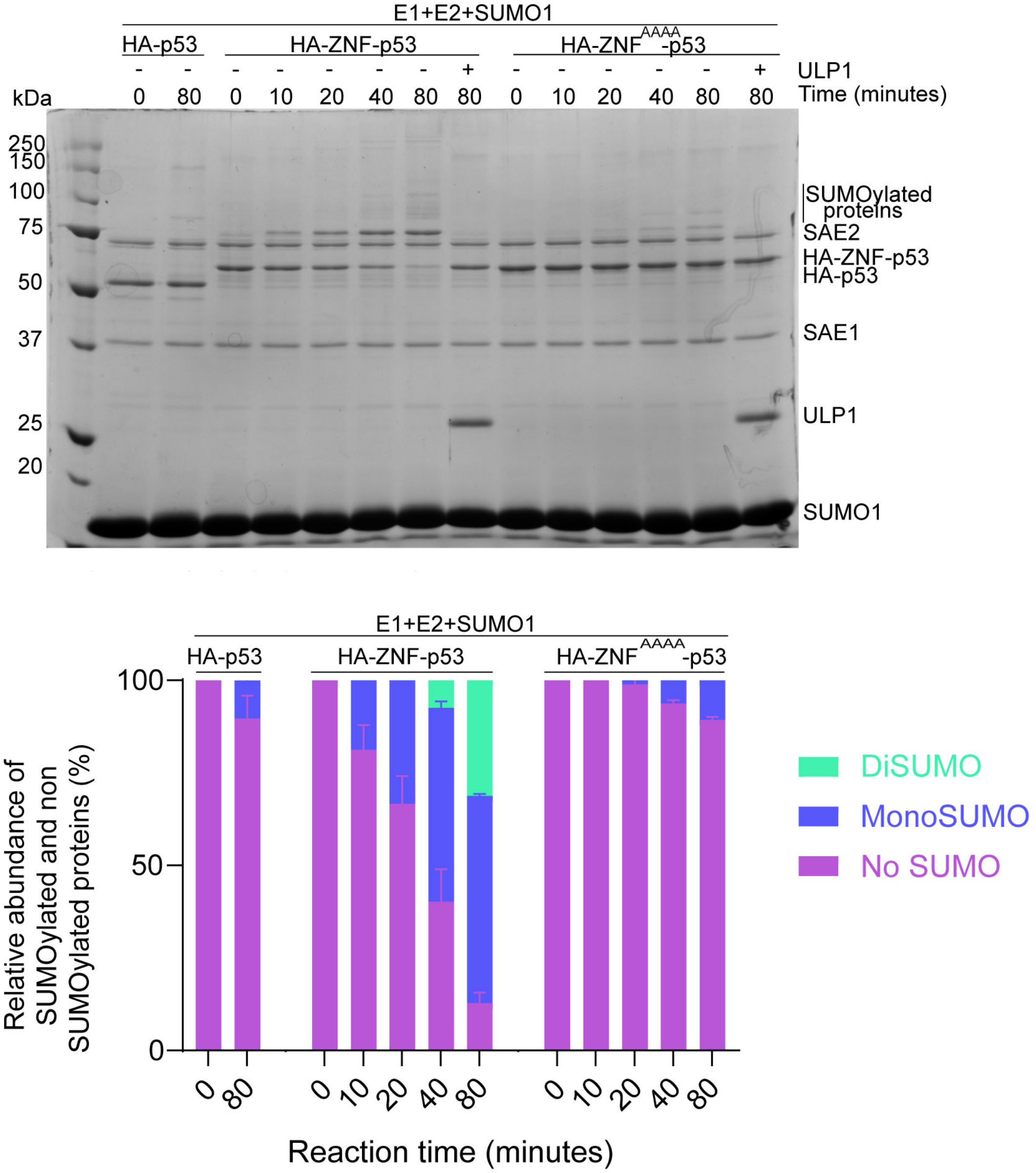

Figure S5: Mutated ZNF SUMO E3 ligase displays less activity with SUMO1 isoform in fusion

(A) *In vitro* assay comparing the SUMOylation of HA-ZNF-p53 and HA-ZNF^{AAAA}-p53 using SUMO1. Reactions were performed and revealed as in Fig. 1. Representative gels from two independent experiments (N=2) are shown. (B) The relative abundance of SUMOylated and unSUMOylated p53 was determined by quantification of band intensities for the conditions presented in (A).

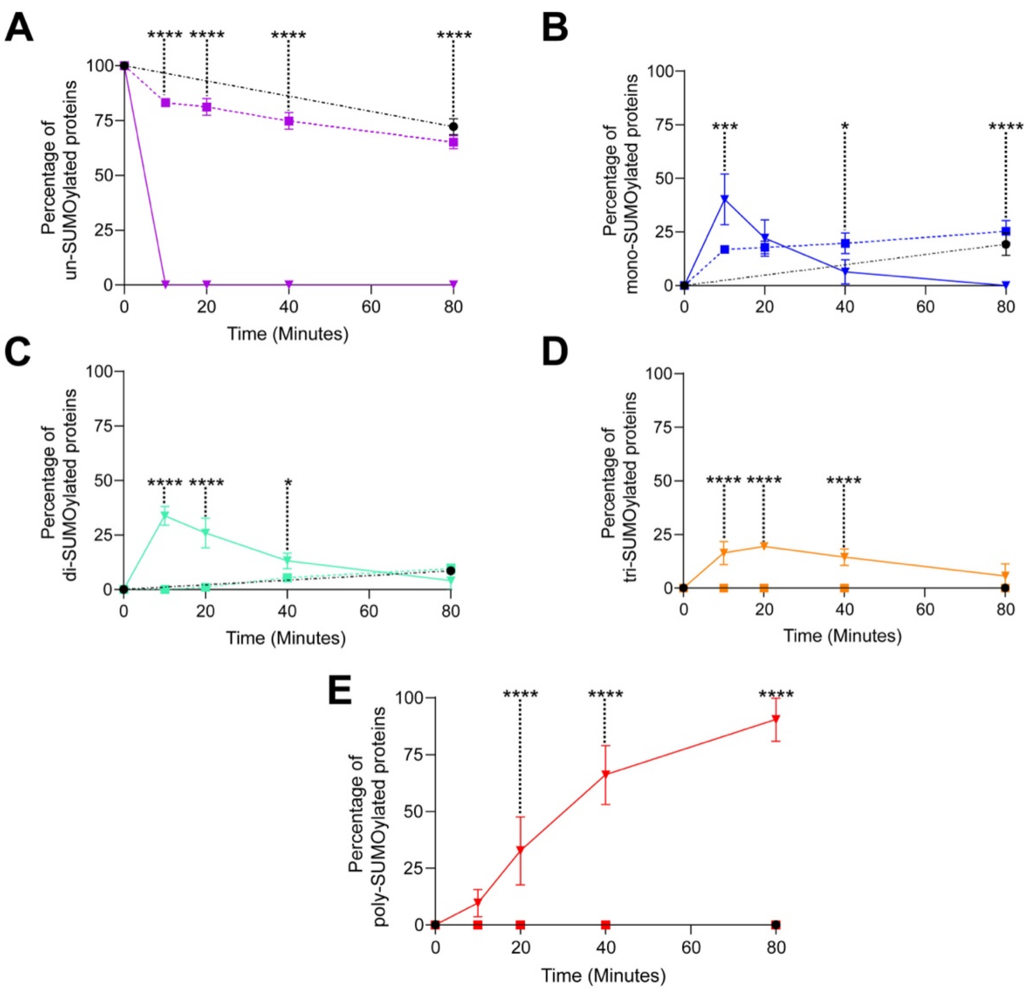

Figure S6: Quantification of SUMOylation associated to the HA-p53, HA-p53 + ZNF, and HA-ZNF-p53 *in vitro* assays with SUMO2 K0

Detailed quantifications from the *in vitro* assays presented in Figure 2E and 2F comparing SUMOylation levels of HA-p53, HA-p53 + ZNF, and HA-ZNF-p53. Relative abundance of (A) un-SUMOylated (B) mono-SUMOylated, (C) di-SUMOylated, (D) tri-SUMOylated, and (E) poly-SUMOylated proteins determined by quantification of band intensities for the conditions presented in Figure 2E. Colors identifying the different stages of SUMOylation are kept the same as Figure 2F. (●) refers to HA-p53, (■) to HA-p53 + ZNF, and (▼) to HA-ZNF-p53. A two-way ANOVA multiple comparisons statistical analysis, where the mean of HA-ZNF-p53 and HA-p53 + ZNF was performed to compare each stage protein modification (*=$p<0.05$, **=$p<0.01$, ***=$p<0.001$, ****=$p<0.0001$). HA-p53 (in black) was excluded from this statistical analysis, and only acts as a reference point

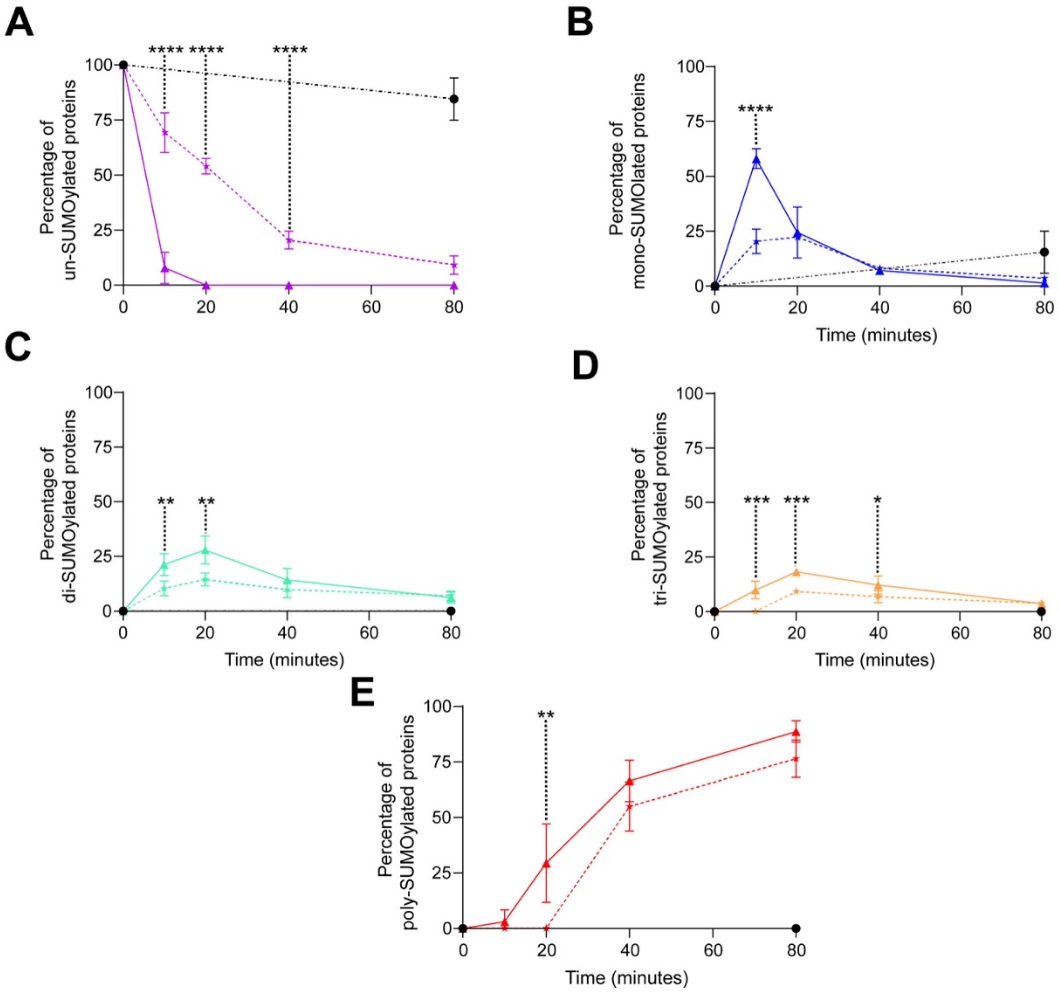

Figure S7: Quantification of SUMOylation associated to the HA-p53, HA-ZNF-p53 + ZNF, and HA-ZNF-p53^{K386R} *in vitro* assays with SUMO2

Detailed quantifications from the *in vitro* assays presented in Figures 3B and 3C comparing SUMOylation levels of HA-p53, HA-p53 + ZNF, and HA-ZNF-p53. Relative abundance of (A) un-SUMOylated (B) mono-SUMOylated, (C) di-SUMOylated, (D) tri-SUMOylated, and (E) poly-SUMOylated proteins determined by quantification of band intensities for the conditions presented in Figure 3B. Colors identifying the different stages of SUMOylation are kept the same as Figure 3C. (●) refers to HA-p53, (▲) to HA-ZNF-p53, and (★) to HA-ZNF-p53^{K386R}. A two-way ANOVA multiple comparisons statistical analysis, where the mean of HA-ZNF-p53 and HA-ZNF-p53^{K386R} was performed to compare each stage protein modification (*=$p<0.05$, **=$p<0.01$, ***=$p<0.001$, ****=$p<0.0001$). HA-p53 (in black) was excluded from this statistical analysis, and only acts as a reference point.

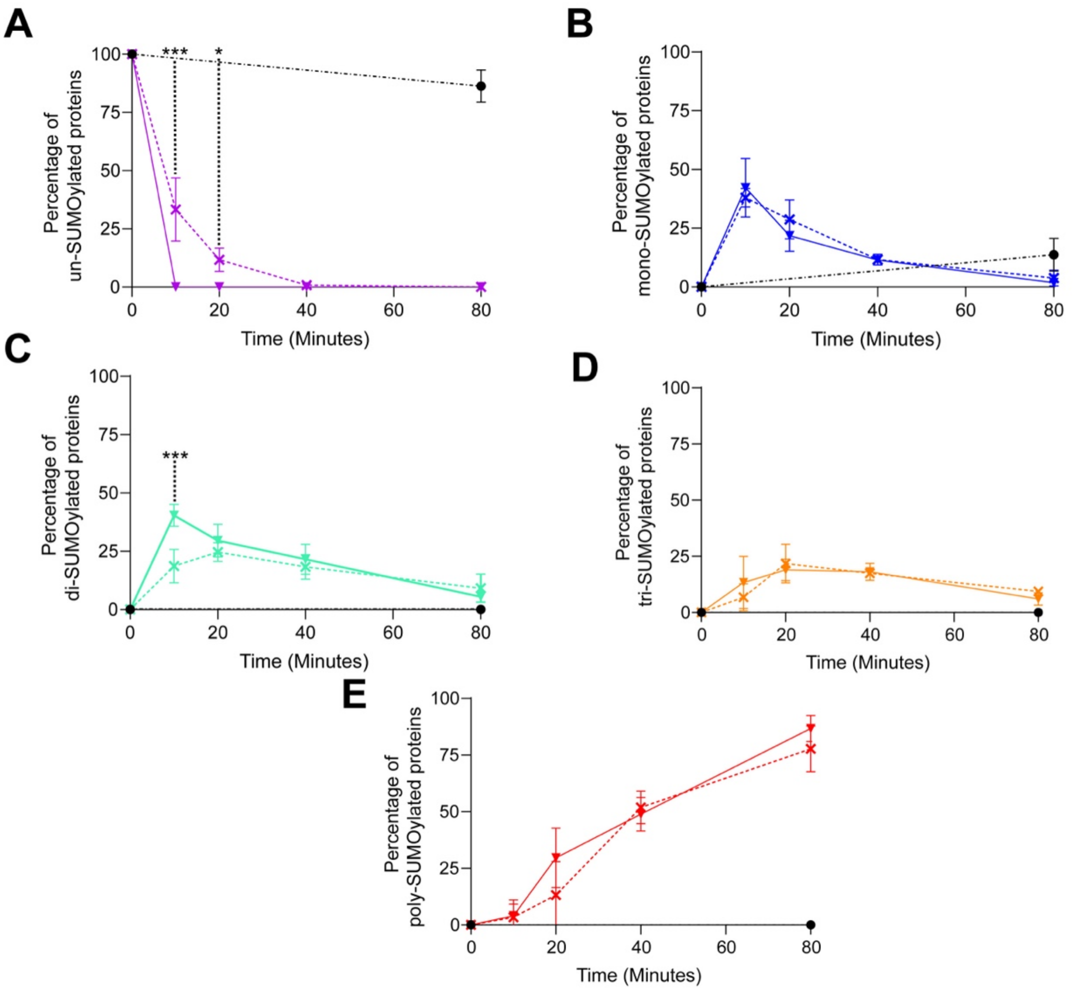

Figure S8: Quantification of SUMOylation associated to the HA-p53, HA-ZNF-p53 + ZNF, and HA-ZNF-p53^{K386R} *in vitro* assays with SUMO2K0

Detailed quantifications from the *in vitro* assays presented in Figures 3E and 3F comparing SUMOylation levels of HA-p53, HA-p53 + ZNF, and HA-ZNF-p53. Relative abundance of (A) un-SUMOylated (B) mono-SUMOylated, (C) di-SUMOylated, (D) tri-SUMOylated, and (E) poly-SUMOylated proteins determined by quantification of band intensities for the conditions presented in Figure 3E. Colors identifying the different stages of SUMOylation are kept the same as Figure 3F. (●) refers to HA-p53, (▼) to HA-ZNF-p53, and (×) to HA-ZNF-p53^{K386R}. A two-way ANOVA multiple comparisons statistical analysis, where the mean of HA-ZNF-p53 and HA-ZNF-p53^{K386R} was performed to compare each stage protein modification (*=$p<0.05$, **=$p<0.01$, ***=$p<0.001$, ****=$p<0.0001$). HA-p53 (in black) was excluded from this statistical analysis, and only acts as a reference point.

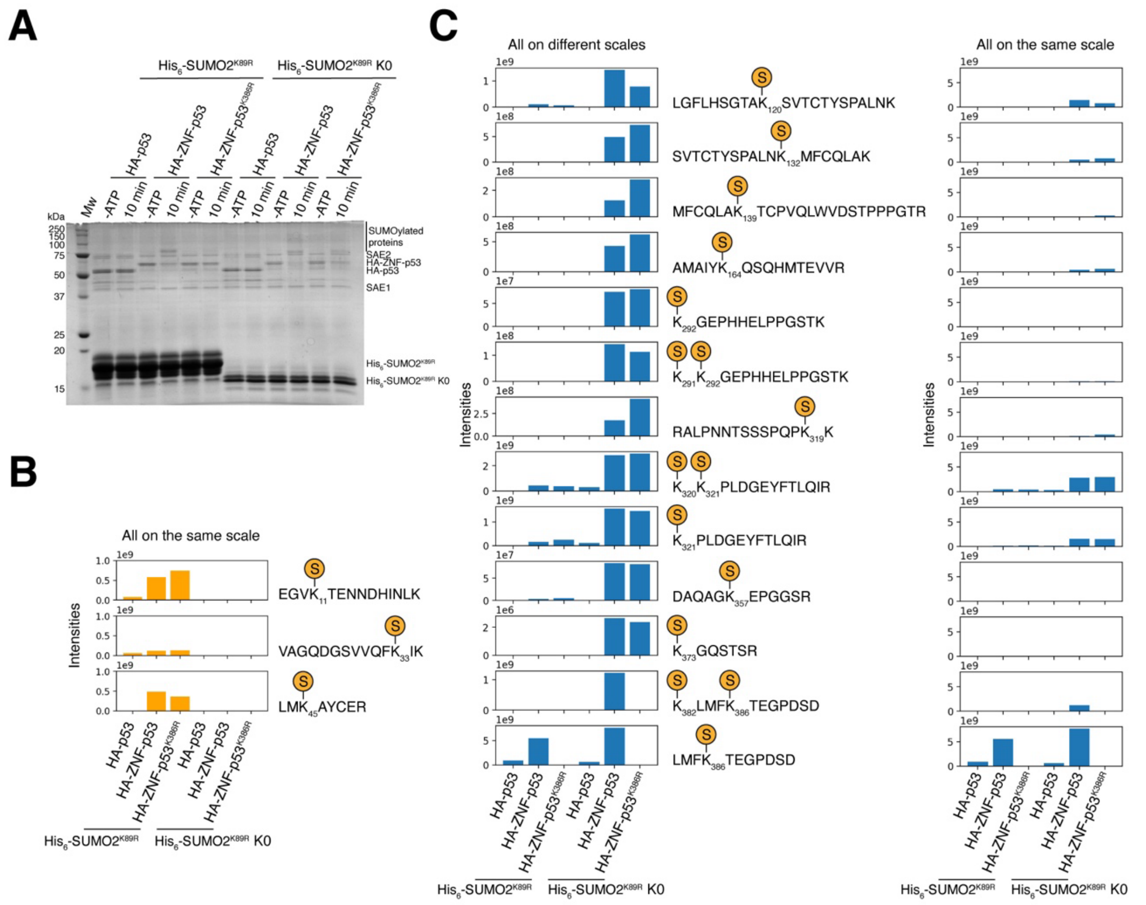

Figure S9: Mass spectrometry analysis of SUMOylation reactions performed *in vitro*.

(A) SDS-PAGE gel colored with Coomassie Blue showing SUMOylation reactions performed using HA-p53, HA-ZNF-p53 or HA-ZNF-p53^{K386R} and either His$_6$-SUMO2^{Q88R} or His$_6$-SUMO2^{Q88R} K0. Samples were taken both before addition of ATP and after 10 minutes of reaction using 5 mM ATP. Only a fraction of the reactions was used for visualization on gel. The rest of the reaction was inactivated by heating to 95°C and analyzed by mass spectrometry. (B) Mass spectrometry analysis of samples presented in (A) and showing the addition of a QQTGG adduct on His$_6$-SUMO2^{Q88R} in presence of either HA-p53, HA-ZNF-p53 or HA-ZNF-p53^{K386R}. The sequences of the identified peptides are presented along with the position of the adducts. (C) Mass spectrometry analysis of samples presented in (A) and showing the addition of a QQTGG adduct on HA-p53, HA-ZNF-p53, or HA-ZNF-p53^{K386R}. The sequences of the identified peptides are presented along with the position of the adducts.

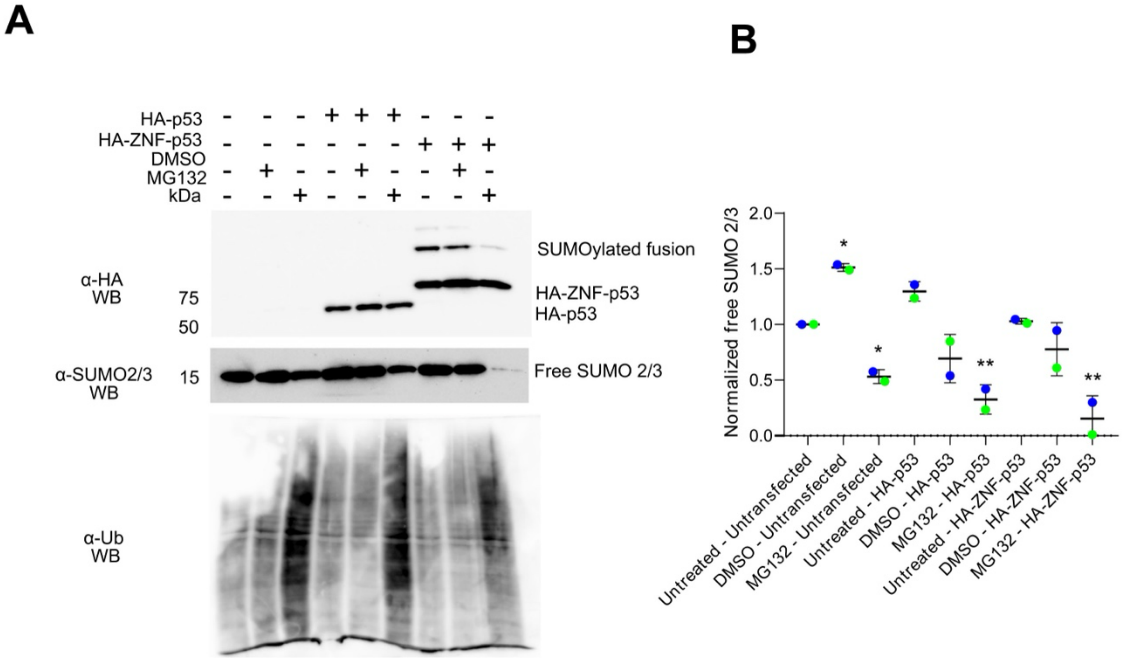

Figure S10: Free SUMO2/3 is depleted following MG132 treatments

(A) Immunoblot analysis of whole cell extracts of HEK293 cells, either untransfected or transfected with HA-p53 or HA-ZNF-p53 for 48h followed by no treatment (untreated) treatment with MG132 or control (DMSO 0.01%) for 4 h. Proteins were separated on a 7.5% or 15% SDS-Page and immunoblots were performed using anti-HA, anti-SUMO2/3, or anti Ub antibodies. Representative immunoblots from two independent experiments (N=2) are shown. (B) Normalized SUMO levels were obtained by dividing the SUMO2/3 band intensity of untreated, untransfected cells to the band intensity of the other conditions. A two-way ANOVA statistical analysis, where each condition was compared to untreated untransfected cells was conducted (*=p<0.05, **=p<0.01, ***=p<0.001, ****=p<0.0001).

A

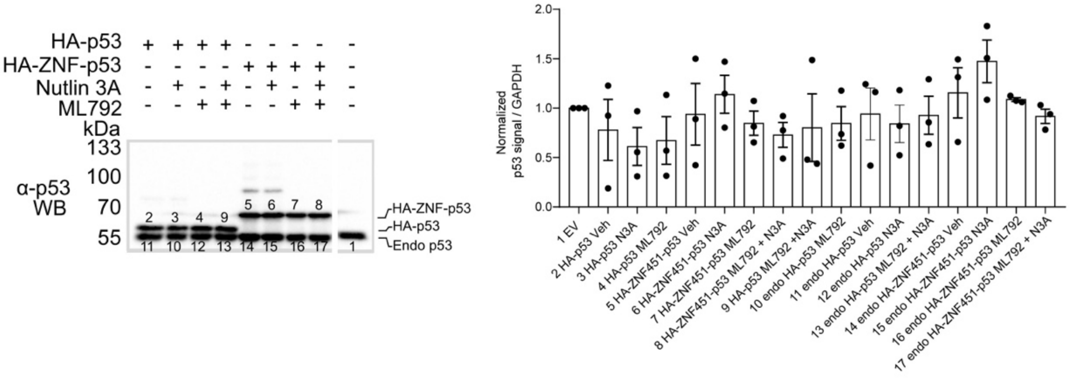

B

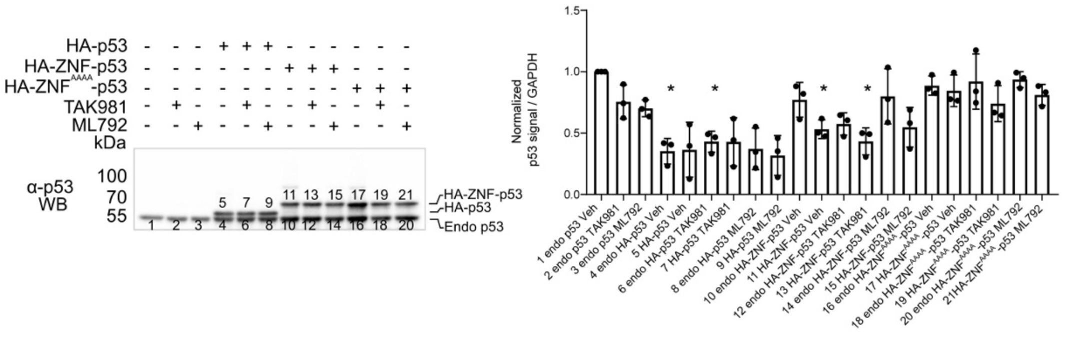

Figure S11: Quantification of endogenous and transfected p53 in the immunoblots associated with the luciferase assays. (A) (B) Levels of endogenous (endo) or transfected p53 in untransfected cells or cells transfected with either HA-p53, HA-ZNF-p53 or HA-ZNF^{AAAA}-p53 that were treated with DMSO, Nutlin 3A, TAK981 or ML792. The quantification refers to the immunoblots presented in Figures 6B and 6D that are duplicated here. The numbers over or under the bands refer to the order they are in the histograms. The ratio intensity corresponds to the p53 band intensity divided by the GAPDH band intensity. Normality was assessed using the Shapiro-Wilk test. A one-way ANOVA statistical analysis followed by Dunnett's multiple comparisons test were performed to determine the effects of the transfected protein or treatment. *=p<0.05, **=p<0.01, ***=p<0.001, ****=p<0.0001.
